# Establishment and application of a vesicle extraction method for clinical strains of *Pseudomonas aeruginosa*

**DOI:** 10.1128/spectrum.03746-25

**Published:** 2026-05-12

**Authors:** Tania Henriquez, Francesco Santoro, Lorenzo Colombini, Donata Medaglini, Lucia Pallecchi, Saioa Sanz, Ilaria Clemente, Agnese Magnani, Eugenio Paccagnini, Mariangela Gentile, Pietro Lupetti, Serena Schwenkert, Chiara Falciani

**Affiliations:** 1 Department of Medical Biotechnologies, University of Siena 574285, Siena, Italy; 2 Department of Biotechnology, Chemistry and Pharmacy, University of Siena 60264, Siena, Italy; 3 Department of Life Sciences, University of Siena 165399, Siena, Italy; 4 Mass Spectrometry of Biomolecules (MSBioLMU), LMU Munich 9183 , Planegg-Martinsried, Germany; Université de Lorraine, Nancy, France

**Keywords:** extracellular vesicles, *Pseudomonas aeruginosa*, vesicle extraction

## Abstract

*Pseudomonas aeruginosa* is a versatile pathogen capable of causing illnesses that range from mild infections to life-threatening conditions. Its virulence is driven by a wide array of factors, among which extracellular vesicles (EVs) have gained recognition as important contributors to pathogenicity. Despite this, the full scope of their roles remains unclear. A major barrier to EV characterization is the difficulty of vesicle isolation—procedures are often lengthy, yield is low, and specialized equipment is required. In this study, we assessed the effectiveness of a rapid vesicle extraction method from clinical strains of *P. aeruginosa*. To that end, we first selected and characterized six phenotypically diverse clinical strains of *P. aeruginosa* (two reference strains and four clinical isolates, including one strain from a cystic fibrosis patient) and used them to evaluate the vesicle extraction method. The results obtained through SDS-PAGE analysis, Western blot, protein quantification, TEM, dynamic light scattering, and mass spectrometry indicated the presence of vesicles in all samples; however, it was also possible to observe a large number of contaminants in some of them (mainly strains LS07 and Z37). Subsequent enzymatic treatments (DNase and/or alginate lyase) allowed for the elimination of these contaminants, as observed by electron microscopy. Our results suggest that this method is suitable for vesicle extraction from clinical isolates of *P. aeruginosa*, where traditional methods, such as ultracentrifugation, are not available. However, the phenotypic complexity of these strains presents challenges that current rapid purification methods are ill-equipped to handle, highlighting the need for improved or alternative approaches.

**IMPORTANCE:**

*Pseudomonas aeruginosa* is a highly adaptable pathogen, and its extracellular vesicles (EVs) play important yet still not fully understood roles in its virulence. Efficient EV characterization depends on reliable extraction/isolation methods, but current approaches are often slow, low-yielding, and require specialized equipment. This study evaluated a new vesicle extraction method using six phenotypically diverse clinical strains. While vesicles were detected in all samples, some preparations contained contaminants, which were successfully reduced through enzymatic treatments. These findings indicate that the method can be used to extract EVs from clinical isolates, allowing these samples to be used for functional screening. With this approach, only preparations with relevant activities could then be purified using conventional protocols (such as density gradient ultracentrifugation), if required.

## INTRODUCTION

*Pseudomonas aeruginosa* is a gram-negative rod-shaped bacterium that can act as an opportunistic pathogen in humans and animals (1–3). This microorganism causes a broad spectrum of diseases, ranging from relatively benign superficial infections—such as those affecting the skin or external ear—to severe and potentially fatal systemic conditions, including pneumonia, bacteremia, and sepsis (1–4). Its pathogenicity is particularly pronounced in immunocompromised individuals, such as patients with cystic fibrosis, burn wounds, or those undergoing invasive medical procedures or immunosuppressive therapy (1–4).

The virulence of *P. aeruginosa* is driven by different factors, such as enzymes, efflux pumps, and extracellular vesicles (EVs). EVs, produced from the bacterial membrane(s), play a critical role in spreading virulence factors (5, 6). These vesicles are enriched with a diverse array of bioactive molecules, including proteins, lipids, DNA, RNA, and virulence factors such as exotoxins and enzymes. EVs play different roles in the pathophysiology of *P. aeruginosa* infections by mediating intercellular communication, facilitating horizontal gene transfer, modulating host immune responses, and enhancing bacterial survival and colonization in hostile environments. The unique properties of *P. aeruginosa*-derived EVs have stimulated interest in their potential biotechnological and therapeutic applications (5, 6). Despite these promising developments, several technical challenges hinder the broader application of EVs in both research and clinical contexts. First, their isolation and purification are difficult because of low yields, contamination with non-vesicular material, and high production costs (7). These limitations underscore the need for the development of standardized, scalable, and cost-effective protocols for EV production and characterization.

In addition, it has been observed that clinical strains of *P. aeruginosa*, which are characterized by high phenotypic diversity (8–11), also possess vesicles with diverse protein content and banding patterns on SDS-PAGE gels (12), suggesting that some isolates may be more active than others or more effective as a biotechnological tool. In this context, there is a need to better characterize the wide variety of EVs from clinical strains. However, to the best of our knowledge, there are currently no extraction methods that can be easily applied in a routine clinical laboratory (without an ultracentrifuge), adapted to the clinical phenotypes of *P. aeruginosa*, and that allow for the easy use of vesicles for screening studies.

In recent years, several rapid methodologies, including commercially available extraction kits, have been developed to streamline and simplify laboratory workflows. These methods are designed to enhance efficiency by reducing hands-on time and improving the overall purity of the extracted material. Despite these advantages, the applicability of such rapid protocols can be limited when working with clinical isolates of *P. aeruginosa* (13).

Therefore, we assessed the effectiveness of a rapid vesicle extraction method for clinical strains of *P. aeruginosa,* compared with ultracentrifugation. We also analyzed the utility of some treatments to improve the quality of the crude extract of our vesicle samples.

## MATERIALS AND METHODS

### Bacterial strains and culture media

*P. aeruginosa* strains were grown in King’s B (KB) medium and maintained on cetrimide agar plates (#70887, Millipore, prepared according to the manufacturer’s instructions). For long-term storage, they were kept in glycerol stocks at −80°C. A complete list of the strains used in this study can be found in Table 1. All strains were cultured at 37°C, unless otherwise specified. King’s B medium (prepared as previously described [14]) was used to grow the strains for vesicle isolation.

**TABLE 1:** List of strains used in this study

Name	Origin (sample type)	Reference
PAO1	Wound	(15)
ATCC27853	Blood	(16)
LS03	Sputum	(13)
LS06	Urine	(13)
LS07	Cerebrospinal fluid	(13)
Z37	Sputum (cystic fibrosis patient)	(17)

### Vesicle isolation

**Fig 1:**
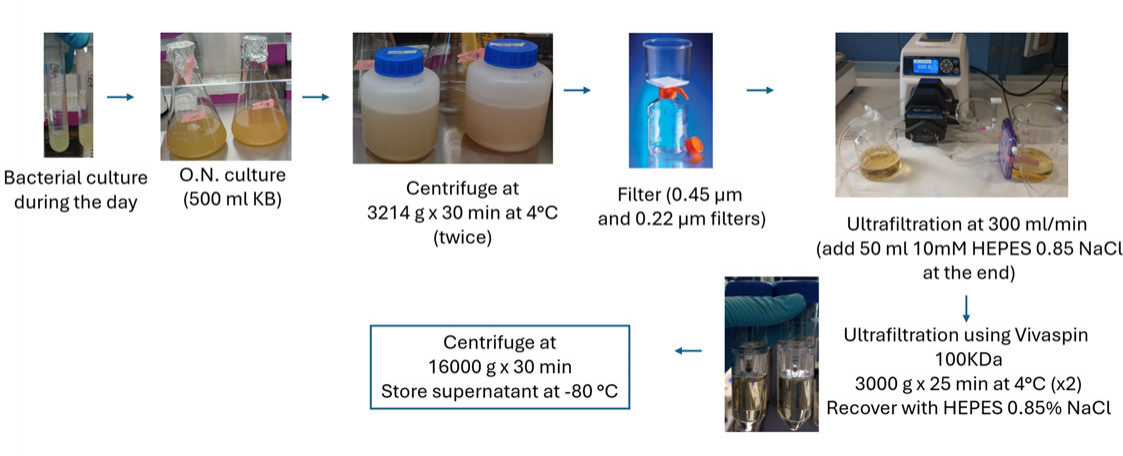
Vesicle extraction method.

Vesicle extraction method using tangential flow filtration (TFF) and ultrafiltration (Fig. 1): Strains were grown in 5 mL of KB medium at 37°C with continuous shaking for 5 h and used to inoculate 500 mL of KB medium. The cultures were incubated overnight at 37°C with continuous shaking until reaching an OD_600_ of 0.79–4.7. Later, the cultures were centrifuged twice at 3,214 × *g* for 30 min at 4°C using Eppendorf 5810R centrifuge (rotor S-4-104 with round buckets and 750 mL bottles), and supernatants were passed through 0.45 and 0.22 µm filters. TFF cassettes (Vivaflow SU, 100 kDa, Sartorius, VF-S050H0100-IV) were used to concentrate the samples. Briefly, ~470 mL of filtrate was concentrated using a feed flow rate of 300 mL/min for 30 min (Cole-Parmer Masterflex L/S pump). Next, 30 mL of 10 mM HEPES 0.85% (wt/vol) NaCl was added to the reservoir and filtration continued for 4 min. Finally, 10 mL of 10 mM HEPES 0.85% (wt/vol) NaCl was added and the sample was recovered. In this way, the samples were concentrated between 11 and 20 times (depending on the specific sample and the presence of large particles). Later, 20–40 mL of sample was centrifuged using a Vivaspin 100 kDa device (3,000 × *g* for 25 min at 4°C, swing-out centrifuge) and resuspended in 10 mM HEPES 0.85% (generating a final volume of 1,000–1,500 µL). A final centrifugation was performed to remove flagellar proteins at 16,000 × *g* for 30 min (fixed-angle centrifuge) (18). The samples were aliquoted and stored at −20°C. All samples were processed in triplicate.

In parallel, PAO1 vesicles were extracted by ultracentrifugation. Briefly, filtered culture supernatants of *P. aeruginosa* were transferred into four polyallomer (36 mL each) centrifuge tubes (25 by 89 mm; Beckman Coulter) and ultracentrifuged at ~134,000 *× g* for 1 h at 4°C in an Optima L-90K ultracentrifuge with the SW32 Ti rotor (Beckman Coulter) to pellet the vesicles. After centrifugation, supernatants were carefully removed and the pellets resuspended in 320 µL of 0.85% (wt/vol) NaCl buffer containing 10 mM HEPES-NaOH (pH 6.8). Of this volume, 80 µL was used for mass spectrometry analysis. For density gradient ultracentrifugation, LS06 sample was loaded at the bottom of a discontinuous Optiprep (Sigma-Aldrich, D1556-250ML) gradient consisting of 2.5 mL layers of 45%, 35%, 30%, 25%, 20%, and 15% Optiprep in 0.85% (wt/vol) NaCl containing 10 mM HEPES-NaOH (pH 6.8). Layers were carefully added to avoid mixing. Gradients were ultracentrifuged at ~154,000 × *g* for 3 h at 4°C. Fractions were carefully collected from the top using a Pasteur pipette and transferred into a six-well cell culture plate, distributing approximately 2.5 mL per well.

### Phenotypic characterization of strains

For pigment production (including pyoverdine) and mucoid phenotype detection, strains were streaked on cetrimide and King’s B agar plates, and incubated at room temperature (RT) for 13 days.

### Vesicle proteome analysis

#### Sample preparation

Proteins present in vesicle samples were reduced with 2 mM dithiothreitol (DTT) for 30 min at 37 °C, followed by alkylation with 5 mM iodoacetamide (IAA) for 30 min at room temperature in the dark. Proteins were digested overnight at 37 °C with trypsin at a 1:50 (wt/wt) enzyme-to-substrate ratio. The resulting peptides were desalted using home-made C18 StageTips, as described by Rappsilber et al. (19).

#### Liquid chromatography–tandem mass spectrometry (LC-MS/MS)

Peptides were analyzed on a nanoElute2 LC system (Bruker Daltonics) coupled to a timsTOF HT mass spectrometer (Bruker Daltonics) equipped with a CaptiveSpray nano-electrospray ionization (nano-ESI) source. Chromatographic separation was performed using a PepSep Ultra analytical column (C18, 25 cm × 75 µm, 1.5 µm particle size, Bruker Daltonics). Peptides were eluted with a linear gradient from 5% to 37% acetonitrile (vol/vol) over 30 min at a flow rate of 300 nL/min, with the column maintained at 50°C. Data-dependent acquisition (DDA) was carried out using the “DDA PASEF-standard_1.1.sec_cycletime.m” method with default instrument parameters.

#### Data processing

Raw mass spectrometry data were analyzed using MaxQuant (version 2.4.14.0; 20). Spectra were searched against the *Pseudomonas* reference proteome (UniProt, UP000002438). Default search parameters were used.

### DNA gel electrophoresis

A 2% agarose gel was prepared in 1 × TBE buffer supplemented with 1 × GelStar (Lonza, #50535). Vesicle samples were loaded on the gel (using GelPilot loading dye 5×, QIAGEN, #239901) and electrophoresis was performed at RT for 30 min at 85 V. Finally, a picture of the gel was taken using a 50 MP camera (moto g31(w), XT2173-3).

### SDS-PAGE and Western blot (WB) experiments

Vesicle samples were used to run two identical SDS-PAGE gels in parallel (12% mPAGE precast gels [#MP12W15, Merck] in 1 × MES SDS, 100 V for 60 min). One of the gels was stained with Thermo Scientific Imperial Protein Stain (#24615, Thermo Scientific) for 2 h and destained overnight with water. The second gel was transferred to a nitrocellulose membrane using a Trans-Blot Turbo Mini 0.2 µm Nitrocellulose Transfer Pack (#1704158, Bio-Rad), according to the manufacturer’s instructions. The membrane was then blocked (3% BSA in TBS) and incubated on a rocker shaker for 1 h at RT. After that, primary antibody solution (containing anti-OprF, Thermo Fisher #PA5-117553, diluted 1:2,500 in TBS + 0.05% Tween, 3% BSA) was added and incubated 1 h at RT on a rocker shaker. Later, the solution was removed, and the membrane was washed three times with TBS + 0.05% Tween (10 min each time) on a rocker shaker at RT. A secondary antibody solution (containing goat Anti-Rabbit IgG antibody, Alkaline Phosphatase conjugate, Sigma-Aldrich #AP132A diluted 1:10,000 in TBS + 5% skimmed milk) was added and incubated for 1 h at RT on a rocker shaker. Again, the membrane was washed three times with TBS + 0.05% Tween (10 min each time) on a rocker shaker at RT. Finally, the buffer was discarded, and BCIP/NBT (Sigma-Aldrich #B1911) was added on top of the membrane (according to the manufacturer’s instructions), incubated for 10 min at room temperature and photographed.

### Transmission electron microscopy (TEM)

For electron microscopy analysis, 3 µL of each sample was loaded onto formvar-coated 300 mesh Cu grids (Ted Pella Inc., Redding, CA, USA) for 2 min at RT. After removing the excess of sample with filter paper, 3 µL of 1% uranyl acetate (Polysciences Inc., Warrington, PA, USA) in distilled water was added for 30 s, blotted again with filter paper and air dried. Finally, the samples were analyzed using a Thermo Fisher Scientific Tecnai G2 Spirit 120 kV transmission electron microscope (equipped with a TVIPS TemCam-F216 CMOS camera).

### Protein quantification

For protein quantification, the Bicinchoninic Protein Assay Kit for dilute samples (Euroclone, #EMP015480) was used according to the manufacturer’s instructions. Briefly, the working reagent was prepared by mixing 25 parts of reagent A, 24 parts of reagent B, and 1 part of reagent C. In parallel, a standard curve using BSA as sample was prepared. Later, 150 μL of working reagent was mixed with 150 μL of sample, standard, or blank, and incubated at 37°C for 2 h. Then, the samples were cooled down at RT, absorbance was measured at 562 nm, and a calibration curve was generated using the reading of each standard vs. its concentration (after correcting for the value of the blank). Finally, the protein concentration of each unknown sample was calculated by interpolating the calibration curve.

### Enzymatic digestion

Samples were treated, when needed, with DNase (#EN0521, Thermo Scientific) and/or alginate lyase (#A1603, Sigma-Aldrich). Briefly, for DNase I treatment, 26 µL vesicle sample was digested with 1 µL (1 U) DNase in the presence of 3 µL 1 × reaction buffer with MgCl_2_. The samples were incubated at 37°C for 30 min, and the reaction was stopped at 65°C for 10 min after the addition of 1 µL 50 mM EDTA. Later, the sample was centrifuged using an Amicon Ultra 0.5 mL (100 kDa) filter and recovered with 150 µL 10 mM HEPES 0.85 NaCl. For alginate lyase, the samples were treated according to the manufacturer’s instructions with a minor modification. Briefly, 150 µL of enzyme solution (12 U/mL) was added to 450 µL of vesicle sample (pH 6.3) plus 10 mM HEPES 0.85 NaCl and incubated at 37°C for 30 min. The reaction was terminated by addition of 465 µL of 0.1 N NaOH. According to the manufacturer, one unit of alginate lyase (units/g solid) will produce an increase in A235 of 1.0 per min per mL of sodium alginate substrate solution at pH 6.3 and 37°C. Finally, samples were concentrated with Amicon Ultra 0.5 mL (100 kDa) and recovered with 150 µL 10 mM HEPES 0.85 NaCl.

### Genome sequencing and analysis

For DNA extraction, strains were grown on KB medium at 37°C overnight with continuous shaking and then centrifuged. Cell pellets were used according to the manufacturer’s instructions (#NR27300, Norgen Biotek). DNA quality was tested by agarose gel electrophoresis and spectrophotometry (IMPLEN N60). DNA sequencing was performed by Biomarker Technologies (BMK) GmbH, Germany, using the Illumina platform. Briefly, for genome assembly, the filtered reads were assembled using Spades v3.6.2. Coding genes were predicted by Prodigal v2.6.3. Genome-wide scanning was performed with GenBlastA v1.0.4 after masking predicted functional genes, and putative candidates were further analyzed by searching for non-mature and frameshift mutations using GeneWise v2.2.0. For functional annotation, the predicted proteins were searched against Nr, Swiss-Prot, TrEMBL, KEGG, and EggNOG databases using BLAST (*e*-value cutoff: 1e−5). Gene Ontology (GO) annotation was performed using Blast2GO. A phylogenetic tree was constructed based on these sequences using Q-TREE v1.6.117. Single-copy gene family sequences were aligned using MAFFT v7.2058 (Parameter: –localpair –maxiterate 1000). Poorly aligned regions or regions with large differences were removed using Gblocks v0.91b9 (Parameter: -b5 = h). All aligned gene family sequences of each species were concatenated to obtain the super gene. ModelFinder10 in IQ-TREE was used for model screening to obtain optimal model. This model was used for phylogenetic tree construction by maximum likelihood (ML), where bootstrap was set at 1,000. Data have been deposited under Bioproject accession number PRJNA1440481 in the NCBI BioProject database (https://www.ncbi.nlm.nih.gov/bioproject/).

### Dynamic light scattering (DLS) analysis

Particle size distribution and polydispersity index (PDI) were determined using a Zetasizer Ultra RED system (Malvern, UK) with a backscatter (173°) detector angle. All analyses were performed in triplicate, with mean size (*Z*-average) and PDI, and respective standard deviations (SDs) of the replicates reported. The data were analyzed by ZS Explorer software from Malvern Panalytical (UK), which employs Cumulant analysis. The samples were diluted 1:40 in ultrapure water and analyzed.

For particle concentration analyses, the samples were measured at three detector angles, namely backscatter (173°), side scatter (90°), and forward scatter (13°), after performing buffer counts normalization.

### Bacteriolytic activity test

*Escherichia coli* LC705 and *Staphylococcus aureus* ATCC 27853 were grown for 6 h in 2 mL of KB medium at 37°C with continuous shaking and mixed with sterile 1× PBS agar to generate plates with live non-replicating bacteria. The vesicular extracts were inoculated in the form of 10 µL drops on the surface of the agar and left to dry. Agar plates were incubated overnight at 37°C and checked for inhibition halos/zones.

## RESULTS

### Strain characterization

**Fig 2:**
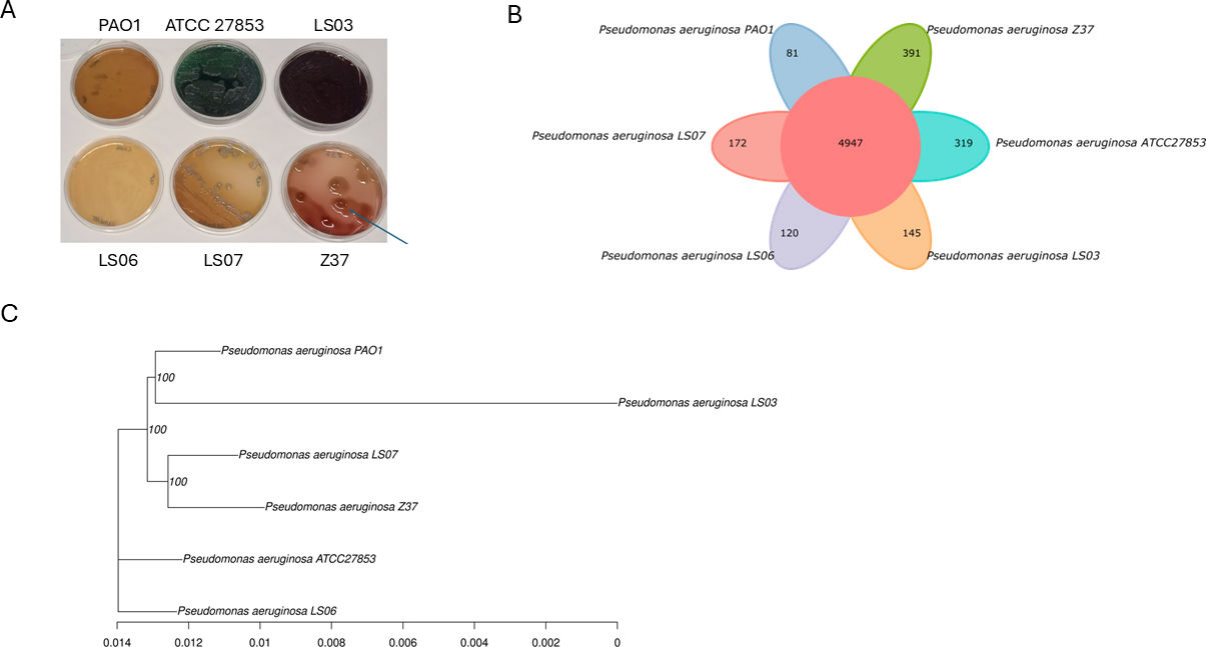
Phenotypic and genetic characterization of clinical strains of *P. aeruginosa*. (**A**) Mucoid phenotype and pigment production analysis. *P. aeruginosa* strains were grown on cetrimide agar and monitored for 13 days at room temperature and photographed. Blue arrow indicates mucoid colony of Z37 strain. (**B**) Venn diagram showing core gene family and unique genes of *P. aeruginosa* clinical strains. (**C**) Phylogenetic tree of clinical strains of *P. aeruginosa*.

We selected six clinical isolates of *P. aeruginosa* (including one strain from a patient with cystic fibrosis, Table 1) from a group of strains that we previously characterized (13, 17). We then analyzed them for pigment production and mucoid phenotype in cetrimide agar. To that end, the strains were grown 13 days at RT on cetrimide agar plates, a selective medium for *Pseudomonas* that induces the production of pigments such as pyoverdine and pyocyanin (21). Our results indicated that after long-term incubation, the strains produced different pigments (Fig. 2A), and only Z37 strain showed a mucoid phenotype on cetrimide agar (Fig. 2A). In order to further characterize the strains and their differences (and complement the previously published analysis regarding antibiotic resistance, growth profiles, pigment production, among others [13]), we sequenced their genomes and analyzed the core genome and dispensable genome. Our results indicated that the core genome consisted of 4,947 genes, while dispensable genes ranged from 81 to 391, depending on the strain (Fig. 2B). Additionally, our whole genome-based phylogenetic tree showed that four genetic groups were formed with these clinical strains (Fig. 2C). These findings reinforce our previous characterization and support the fact that these strains have a largely conserved genome, although they exhibit high phenotypic diversity and are representative of the variability described for clinical strains of *P. aeruginosa* (8, 13).

### Vesicle extraction and characterization

Previously, we tested a commercial kit for the isolation of vesicles from *P. aeruginosa* clinical strains with little success (13). Based on our previous experience, we decided to design a protocol for the extraction of vesicles from clinical isolates. To that end, we adapted protocols reported by other groups for *Pseudomonas* or for other microorganisms (18, 22–24). The rationale for the protocol was to use equipment that is normally present in microbiology laboratories or that can be easily acquired (Fig. 1). In this context, relatively small volumes of culture were preferred as they are much more easily centrifuged. Briefly, an inoculum was grown during the day and used to inoculate a flask containing 500 mL of KB, which was then incubated overnight. The sample was centrifuged and filtered several times (using 0.45 and 0.22 µm filters). At the end of the ultrafiltration, a vesicle crude extract was obtained. Finally, the sample was centrifuged at 16,000 × *g* for 30 min to remove flagellar proteins, as suggested by previous works (18).

**Fig 3:**
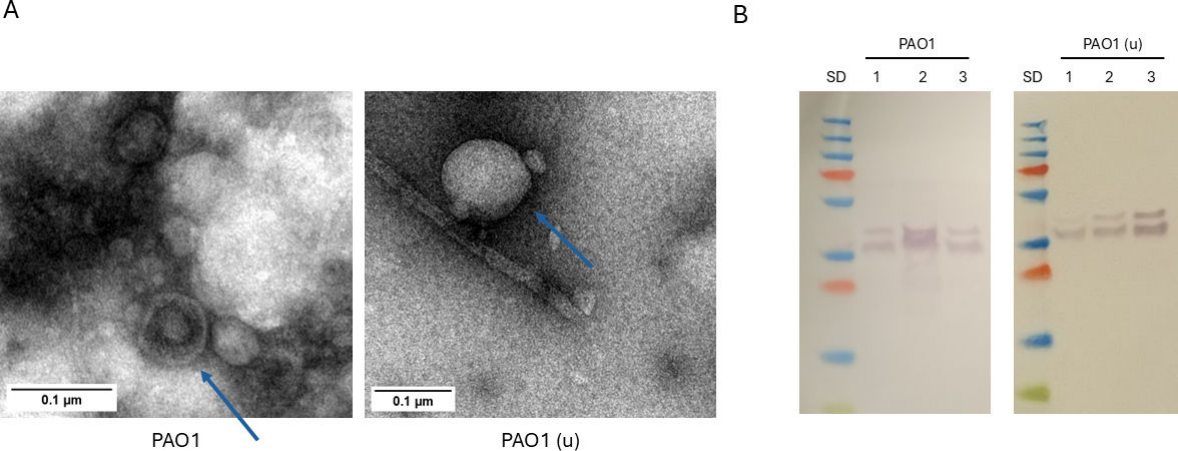
Characterization of PAO1 vesicles extracted with two methods. (**A**) TEM images of vesicles from PAO1 strain extracted by ultrafiltration or by ultracentrifugation (PAO1u). Blue arrows indicate vesicles. (**B**) Western blot for vesicle extracts of PAO1 strain by ultrafiltration or by ultracentrifugation (PAO1u). Western blot was performed using antibody against OprF. SD: PageRuler Prestained Protein Ladder.

Vesicles from the PAO1 strain were isolated using the two different methods, the above protocol and a standard ultracentrifugation-based method used for comparison. The vesicle samples obtained from both methods were analyzed by Western blot (WB) using an antibody against the OprF porin, an important membrane protein of *P. aeruginosa* (25, 26), as well as by transmission electron microscopy (TEM), protein quantification, and dynamic light scattering (DLS). TEM analysis confirmed the presence of vesicles in samples obtained with both isolation methods (Fig. 3A).

When analyzed by Western blot, both samples showed the characteristic signal of the OprF membrane protein (Fig. 3B). Additionally, analysis of the triplicates using DLS indicated that samples extracted with our method were mostly monodisperse (except for the second replicate, Fig. S1), while samples extracted by ultracentrifugation were mostly polydisperse (Fig. S2) and have a comparable *Z*-average. DLS analysis also showed a higher number of particles for PAO1 extracted by ultracentrifugation (1 × 10^11^) compared to our method (1 × 10^9^) (although because the samples are not monodisperse, these values are only estimates). In parallel, protein quantification performed on triplicate samples showed a higher protein concentration in vesicles extracted by ultracentrifugation. Nevertheless, these results indicate that our protocol is capable of isolating vesicles in a manner comparable to the ultracentrifugation method. Based on these findings, vesicles from clinical strains were subsequently isolated using our protocol.

**Fig 4:**
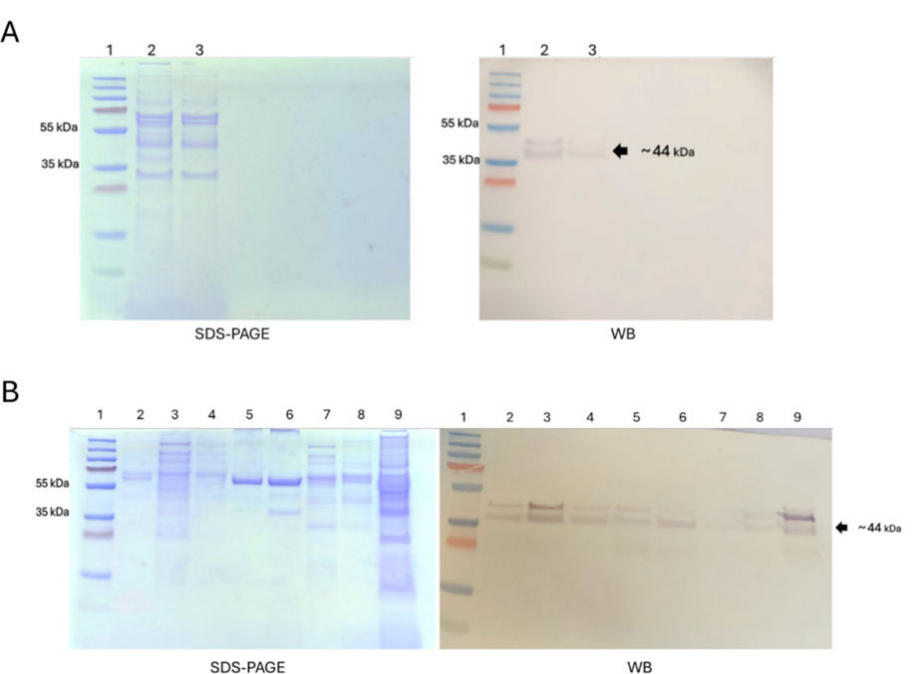
Vesicle analysis. (**A**) Vesicles from PAO1 strain were analyzed by SDS-PAGE (left) and Western blot, WB (right). For SDS-PAGE, Imperial Protein stain was used. For WB, an antibody against OprF porin conjugated with alkaline phosphatase was used. For both images, 1: PageRuler Prestained Protein Ladder; 2: PAO1 vesicles—crude extract; and 3: PAO1 vesicles after last centrifugation to remove flagella. (**B**) Vesicle samples from clinical strains were analyzed by SDS-PAGE (left) and WB (right). For SDS-PAGE, Imperial Protein was used, and for WB, antibody against OprF porin. For both images, 1: PageRuler Prestained Protein Ladder; 2: PAO1; 3: ATCC 27853; 4: LS03; 5: LS06; 6: LS07; 7: Z37; 8: PAO1 (final sample—pilot experiment); and 9: PAO1 pellet (pilot experiment).

**Fig 5:**
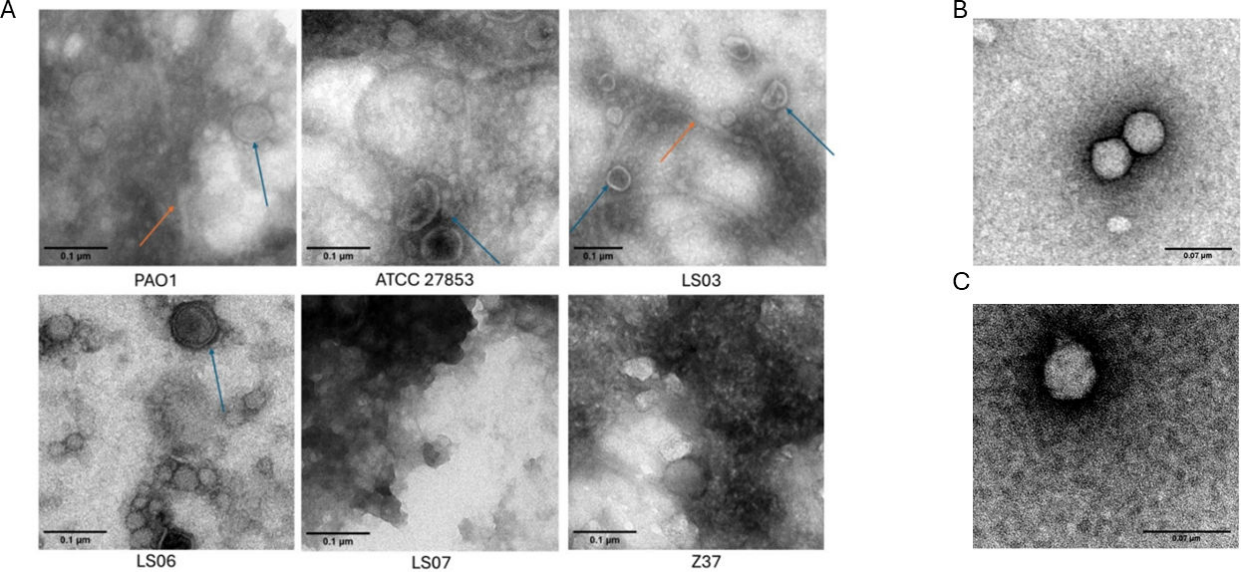
Electron microscopy images of vesicle samples. (**A**) Blue arrows indicate extracellular vesicles, and orange arrows indicate flagellar proteins. Samples LS07 and Z37 showed a high number of unidentified contaminants. (**B and C**) Electron microscopy images of alginate lyase-treated vesicle samples. Samples (**B**) LS07 and (**C**) Z37 were treated with 12 units of alginate lyase for 10 min and analyzed by TEM.

All samples from the different strains were analyzed by SDS-PAGE, Western blot for OprF (Fig. 4A and B), and TEM (Fig. 5). Our results showed that vesicle samples from different strains had distinctly different protein patterns in SDS-PAGE (Fig. 4B), which is in agreement with previous reports (12). This difference in the protein pattern between samples was consistent across triplicates (Fig. S3). All samples were positive for OprF; however, strain Z37 showed a markedly lower signal intensity compared with the other strains.

Mass spectrometry analysis of the samples revealed that all extracts contained the GroEl protein, which is in agreement with previous reports (27) (Data S1). The samples with the highest number of identified proteins were ATCC 27853 and LS03, while the sample with the lowest number of hits was LS07 (Table 2). These results support the difference in protein patterns observed by SDS-PAGE. Several proteins were missing in some aliquots, as the protein concentration in these samples was not optimal for this analysis.

**TABLE 2:** Mass spectrometry results

Sample	Number of proteins identified (triplicate average)
ATCC 27853	45
LS03	44
LS06	12
LS07	1
Z37	16
PAO1	9
PAO1u	3

In parallel, TEM analysis showed vesicles in samples PAO1, ATCC 27853, LS03, and LS06 (Fig. 5). However, the images also showed the presence of flagellar structures, indicating that the final centrifugation step reduced but did not completely eliminate these contaminants. Furthermore, triplicate analyses indicated that most samples were polydisperse (Data S2).

Additionally, vesicles were not clearly observed in samples LS07 and Z37 due to the presence of additional contaminants (Fig. 5). These contaminants may also explain the lower OprF signal detected by WB.

Samples LS07 and Z37 were therefore treated with alginate lyase, as alginate is a major component of the Pseudomonas capsular exopolysaccharide and is associated with the mucoid phenotype (27). Alginate lyase treatment reduced contaminants in sample LS07, allowing clearer visualization of vesicles by TEM (Fig. 5B). In contrast, sample Z37 still contained additional particles not associated with vesicles (data not shown). Agarose gel electrophoresis revealed the presence of genomic DNA and possible plasmid DNA in Z37 (Fig. S1), and the sample was subsequently treated with DNase I. The combined enzymatic treatment improved sample purity, as observed by TEM (Fig. 5C). However, several vesicles appeared damaged or aggregated, suggesting that the extended protocol and enzymatic treatment may affect vesicle integrity. Additionally, the vesicles observed in this sample were, on average, smaller than those detected in the other strains.

Nevertheless, the results indicated that it was possible to clean the samples of most of the interferents observed in the TEM images, suggesting the usefulness of the protocol and the possibility of using these samples for further functional studies.

### Functional study—bacteriolytic activity

In order to assess whether the isolated vesicles retained their functional capabilities, a bacteriolytic activity assay was selected (since it has previously been reported that some vesicles exhibit lytic activity against other species [28]). To that end, strains of *E. coli* and *S. aureus* were cultured and mixed with agar prepared in 1× PBS, to obtain agar plates with non-replicating bacteria. These plates were then exposed to aliquots of the vesicles and evaluated for halo formation after overnight incubation. Our results indicated that the vesicle extracts showed no activity against *E. coli*, while some extracts (LS06, LS07, and Z37) did show activity against *S. aureus* (Fig. S5A). The activity of LS06 was further characterized by confirming the lytic activity in another two replicates (Fig. S5B). Finally, OptiPrep density gradient ultracentrifugation indicated that lytic activity is associated with the 25%, 30%, and 35% fractions (Fig. S5C). These fractions show the strongest OprF signal by Western blot (Fig. S5D) and are therefore enriched in vesicles. Taken together, these results indicate that the vesicles extracted using our protocol can be used for functional analysis screenings, as they do not lose their functional capacities.

## DISCUSSION

The primary objective of this study was to evaluate the utility of a vesicle extraction protocol for clinical isolates of *P. aeruginosa*. Our results demonstrate that the method we used enables the recovery of extracellular vesicles (EVs) suitable for downstream functional analyses and screening assays.

Genomic characterization revealed that most of the genome of these strains is conserved (29), although they exhibit great phenotypic diversity, which is in agreement with previous reports (8, 11). Consistent with this diversity, the proteomic profiles of the corresponding EV preparations displayed substantial variability (12). This observation suggests that phenotypic heterogeneity among strains is reflected at the vesicle level, a phenomenon that has been reported previously. Although a small subset of proteins was shared among EV samples, many proteins were isolate-specific, resulting in limited overlap within the core EV proteome. These findings are consistent with previous studies indicating that the molecular cargo of *P. aeruginosa* EVs is strongly strain-dependent and likely reflects both the genetic background and physiological state of the producing cells (30).

Functional assays further demonstrated that this heterogeneity extends to vesicle-associated biological activities. In particular, differences were observed in bacteriolytic activity among EV preparations. While EVs from three isolates exhibited lytic activity against *S. aureus,* none of the EV samples displayed detectable activity against *E. coli*. This selective bacteriolytic effect suggests that the presence of lytic enzymes or antimicrobial factors within EV cargo varies substantially across isolates.

Collectively, these findings support the hypothesis that EVs derived from different clinical isolates possess distinct molecular compositions and functional properties.

The protocol presented in this study enables the extraction of vesicles from clinical strains; however, transmission electron microscopy (TEM) analysis indicated that these vesicles exhibit greater structural damage compared with vesicles obtained through ultracentrifugation (possibly due to the pressure exerted in the ultrafiltration steps). These findings suggest that, while the protocol represents a useful alternative in settings where traditional purification methods are unavailable, it does not match ultracentrifugation in terms of sample quality.

In summary, the phenotypic complexity of these strains presents challenges that conventional purification methods are unable to address. In this context, it is expected that the method designed in this study will aid in the rapid and efficient extraction of EVs in clinical laboratories where traditional methods are not available, allowing these samples to be used for functional screening. In this way, only preparations with relevant activities could then be purified using conventional protocols (such as density gradient ultracentrifugation), if required. In conclusion, this protocol is expected to support further investigations into how distinct vesicle populations derived from clinical *P. aeruginosa* strains contribute to bacterial physiology and pathogenicity.

## Data Availability

The mass spectrometry proteomics data have been deposited to the ProteomeXchange Consortium via the PRIDE partner repository with the data set identifier PXD077187.

## SUPPLEMENTAL MATERIAL

Data S1 spectrum.03746-25-s0001.xlsx Mass spectrometry data.

Data S2 spectrum.03746-25-s0002.pdf DLS data.

Supplemental figures spectrum.03746-25-s0003.docx Fig. S1 to S5.

ASM does not own the copyrights to Supplemental Material that may be linked to, or accessed through, an article. The authors have granted ASM a non-exclusive, world-wide license to publish the Supplemental Material files. Please contact the corresponding author directly for reuse.
