# Supplementary material for: Establishment and application of a vesicle extraction method for clinical strains of Pseudomonas aeruginosa
Source: Microbiol Spectr. 2026 May 12;14(6):e03746-25. doi: 10.1128/spectrum.03746-25 (PMC13227995; doi:10.1128/spectrum.03746-25)
Supplement: Data S2 — DLS data. [file spectrum.03746-25-s0002.pdf]

Supplementary material 3: Dynamic Light Scattering (DLS) data. Analysis of vesicle purification (triplicate samples) by DLS. A correlogram (left) and a graph indicating size distribution by intensity (right) are shown for each set, including a table with the specific data provided by the instrument (Zetasizer).

ATCC 27853

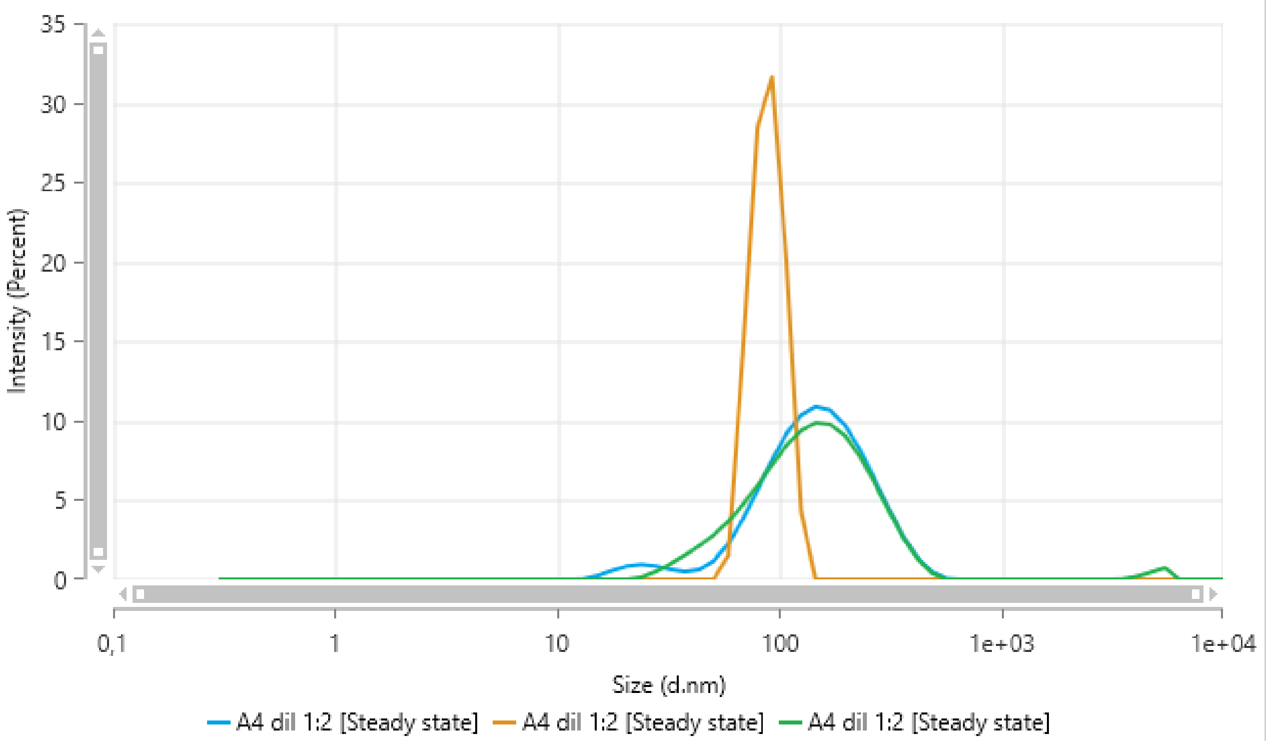

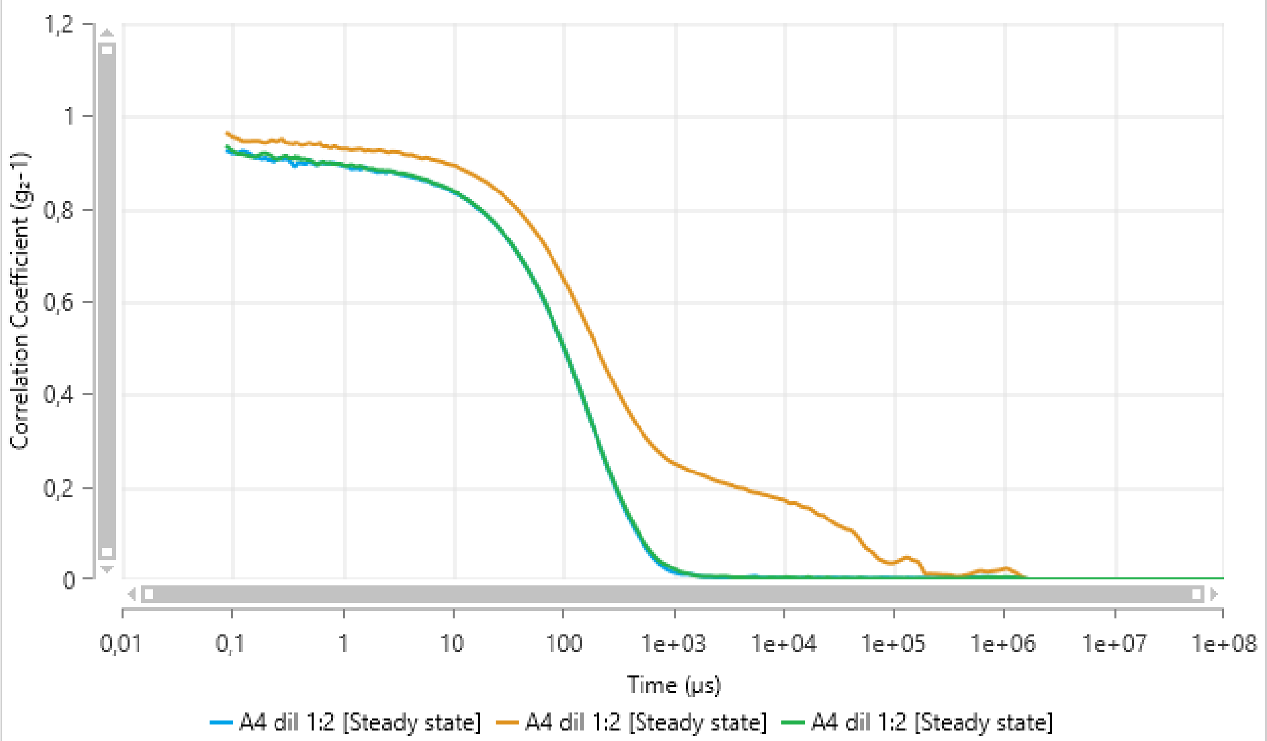

Statistics Table

Name	Mean	Standard Deviation	RSD	Minimum	Maximum
Z-Average (nm)	183	114,2	62,4	115,8	314,8
Polydispersity Index (PI)	0,3638	0,1129	31,03	0,2661	0,4874
Peak 1 Mean by Intensity ordered by area (nm)	138,1	42,25	30,6	89,69	167,6
Peak 1 Area by Intensity ordered by area (%)	98,03	2,327	2,374	95,46	100
Peak 2 Mean by Intensity ordered by area (nm)	2511	3515	140	25,37	4996
Peak 2 Area by Intensity ordered by area (%)	2,96	2,234	75,47	1,38	4,539

ATCC 27853

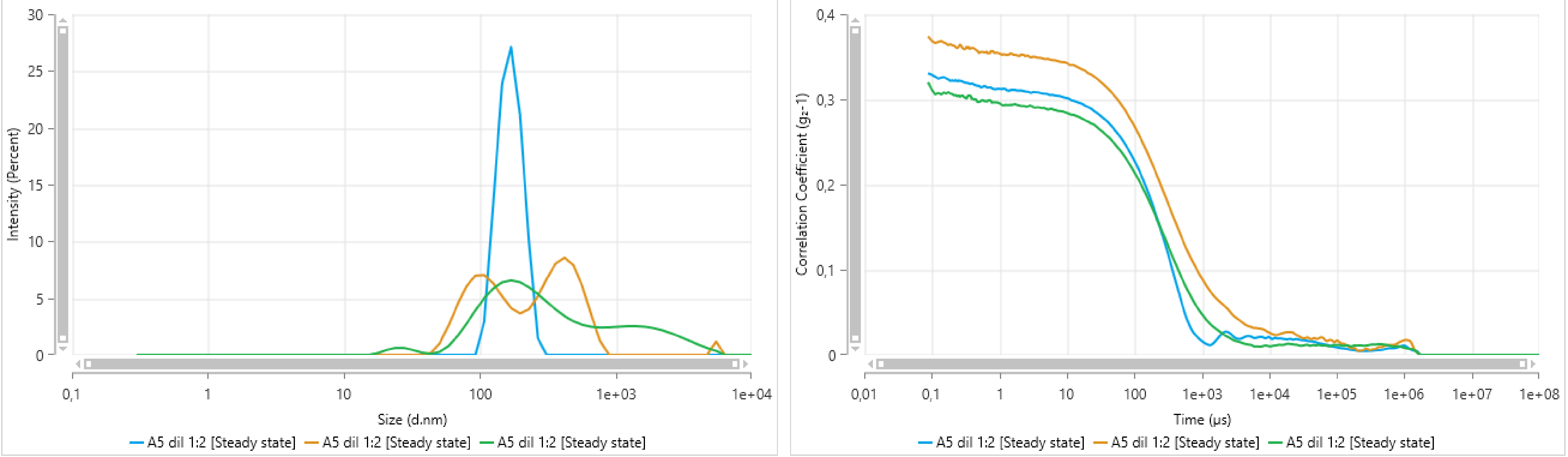

Statistics Table

Name	Mean	Standard Deviation	RSD	Minimum	Maximum
Z-Average (nm)	260,7	89,29	34,25	205,7	363,8
Polydispersity Index (PI)	0,3659	0,1104	30,17	0,2559	0,4767
Peak 1 Mean by Intensity ordered by area (nm)	277,1	118,5	42,77	169,8	404,3
Peak 1 Area by Intensity ordered by area (%)	74,97	23,68	31,59	52,91	100
Peak 2 Mean by Intensity ordered by area (nm)	1001	1255	125,4	113,1	1888
Peak 2 Area by Intensity ordered by area (%)	35,71	14,47	40,53	25,48	45,94

ATCC 27853

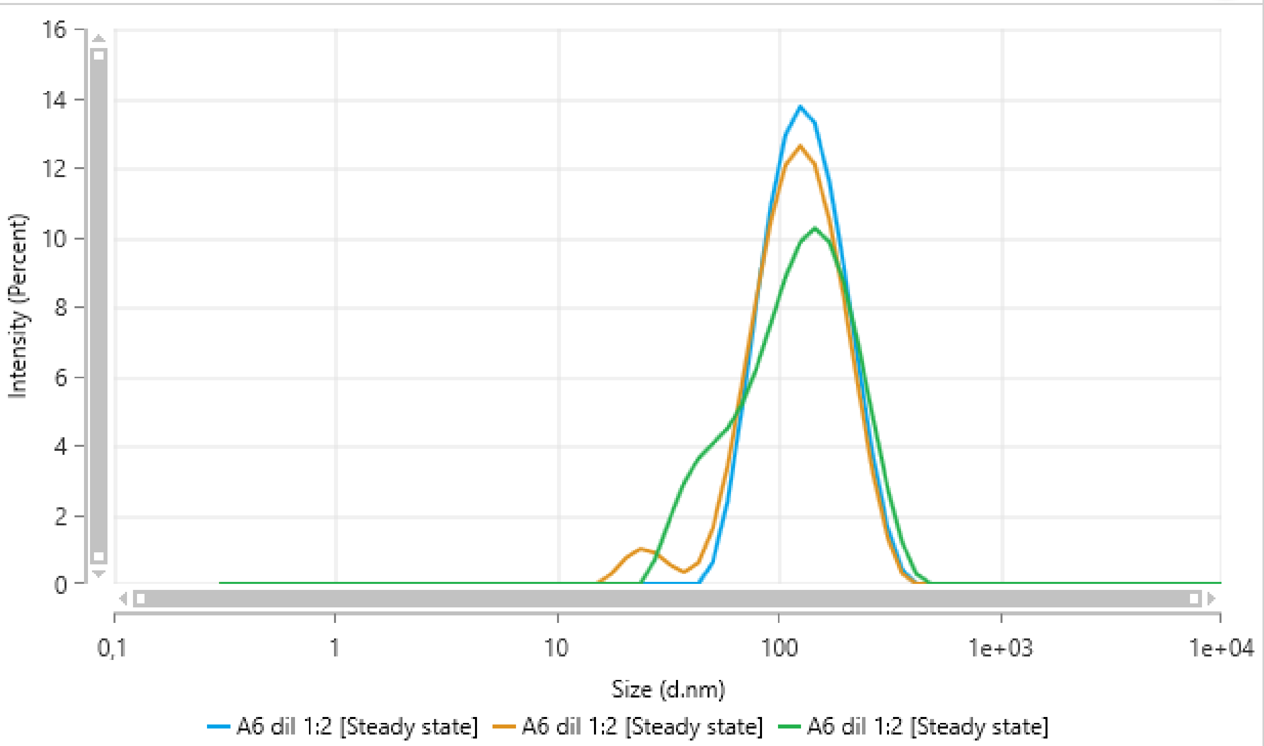

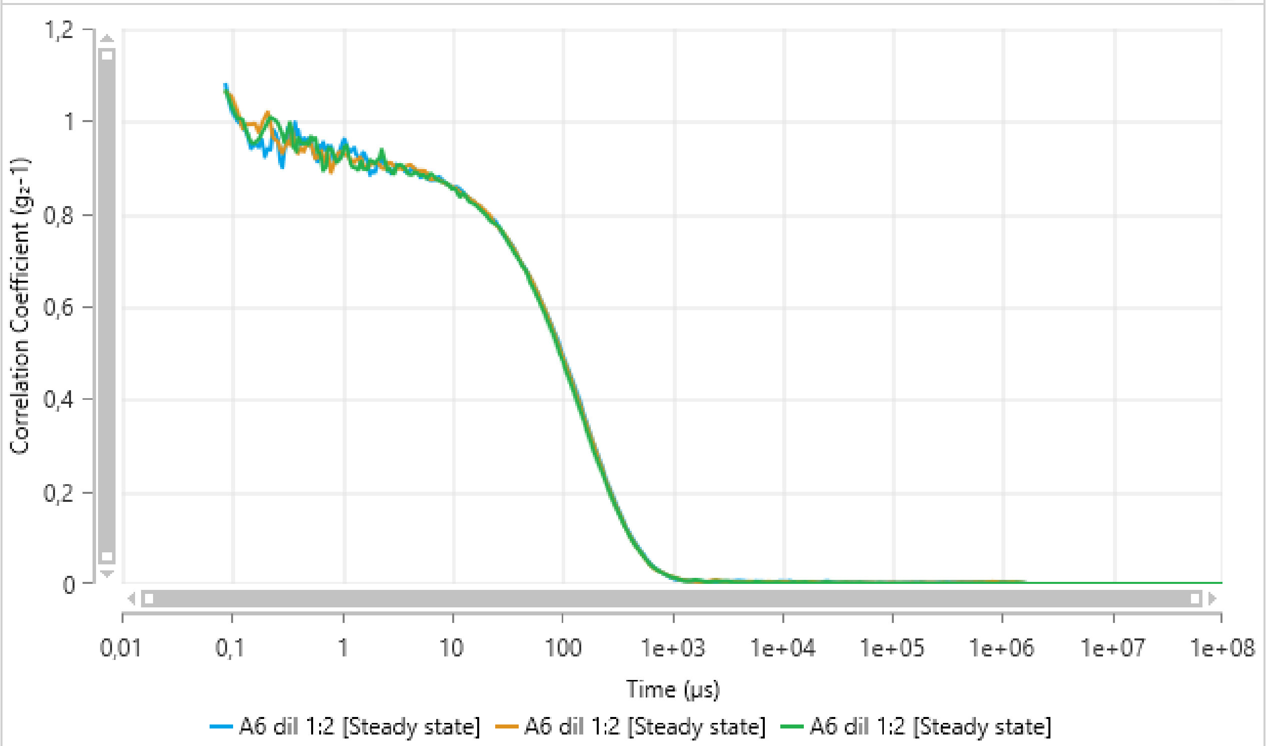

Statistics Table

Name	Mean	Standard Deviation	RSD	Minimum	Maximum
Z-Average (nm)	106,8	3,213	3,008	103,4	109,8
Polydispersity Index (PI)	0,2608	0,01468	5,628	0,2489	0,2772
Peak 1 Mean by Intensity ordered by area (nm)	138,3	2,932	2,119	135,9	141,6
Peak 1 Area by Intensity ordered by area (%)	98,72	2,222	2,251	96,15	100
Peak 2 Mean by Intensity ordered by area (nm)	26,11	-	-	26,11	26,11
Peak 2 Area by Intensity ordered by area (%)	3,848	-	-	3,848	3,848

LS03

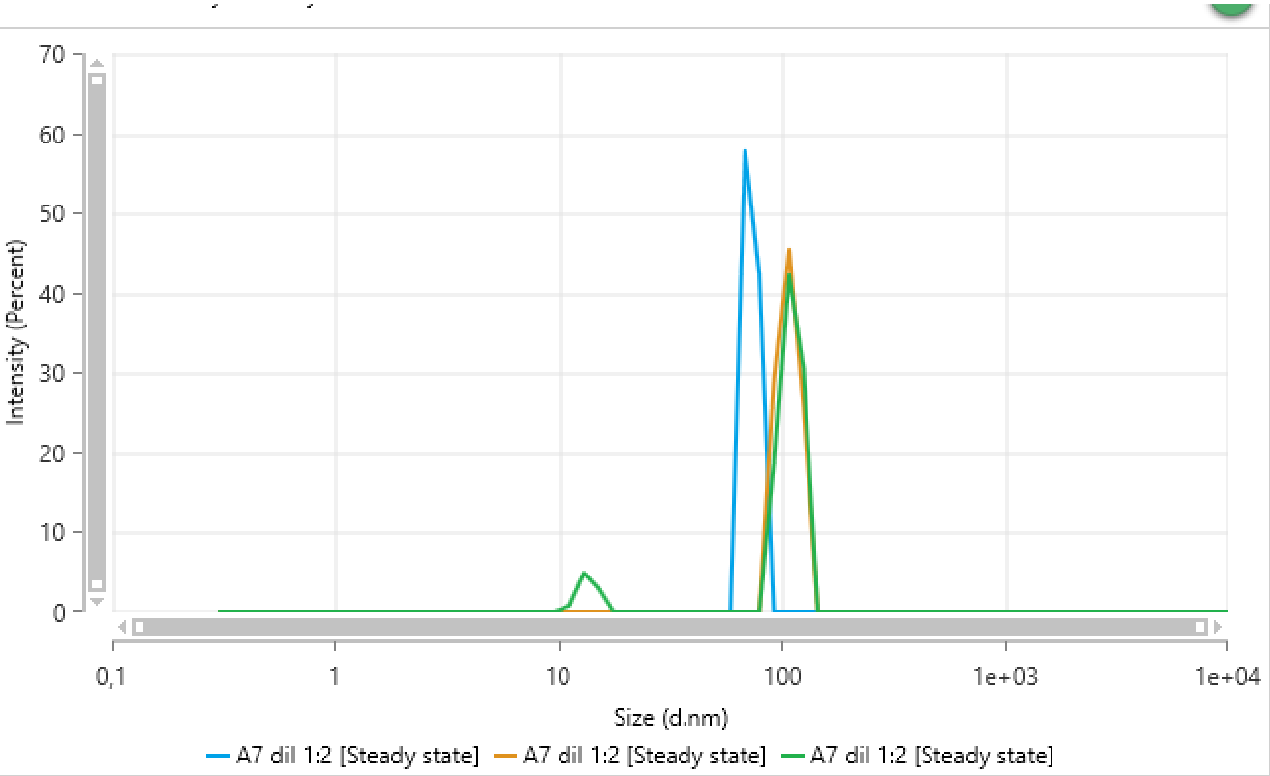

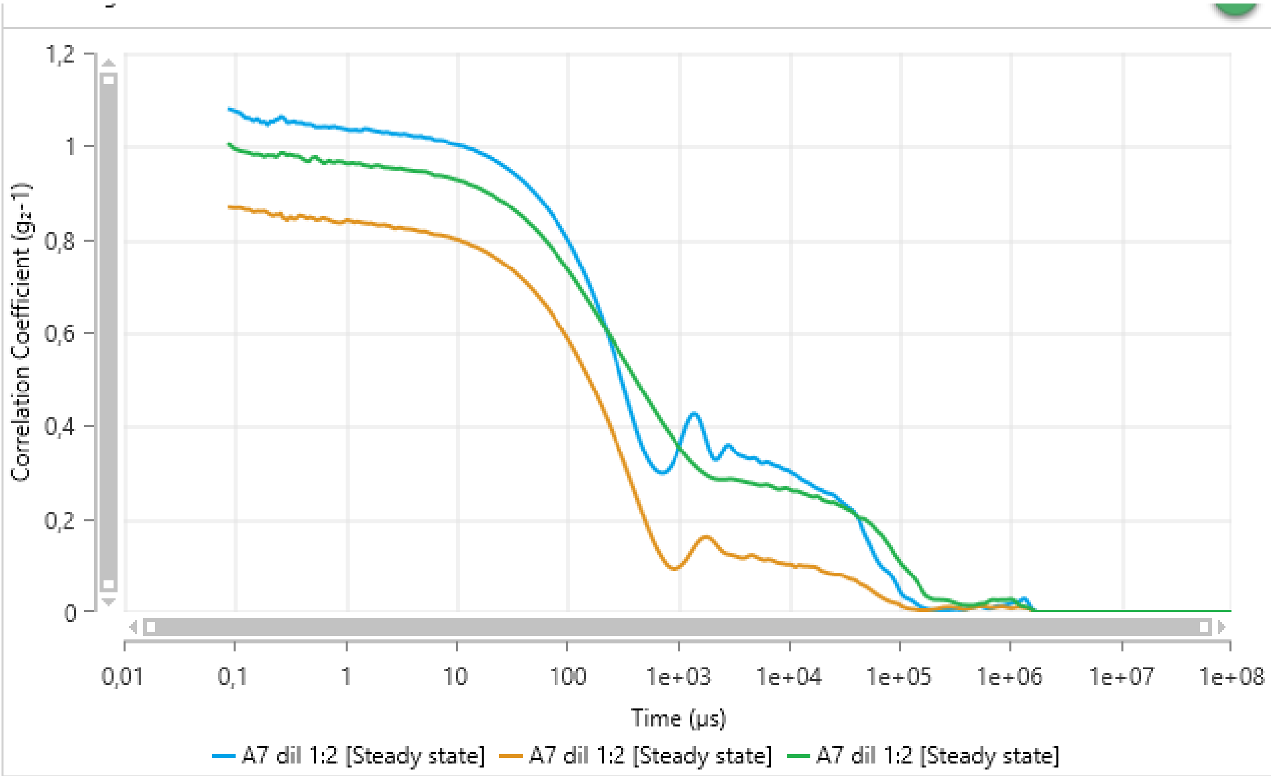

Statistics Table

Name	Mean	Standard Deviation	RSD	Minimum	Maximum
Z-Average (nm)	535,5	211,7	39,53	291,7	672,9
Polydispersity Index (PI)	0,525	0,1468	27,96	0,379	0,6726
Peak 1 Mean by Intensity ordered by area (nm)	97,46	20,86	21,41	73,42	110,8
Peak 1 Area by Intensity ordered by area (%)	97,14	4,957	5,103	91,41	100
Peak 2 Mean by Intensity ordered by area (nm)	13,62	-	-	13,62	13,62
Peak 2 Area by Intensity ordered by area (%)	8,586	-	-	8,586	8,586

LS03

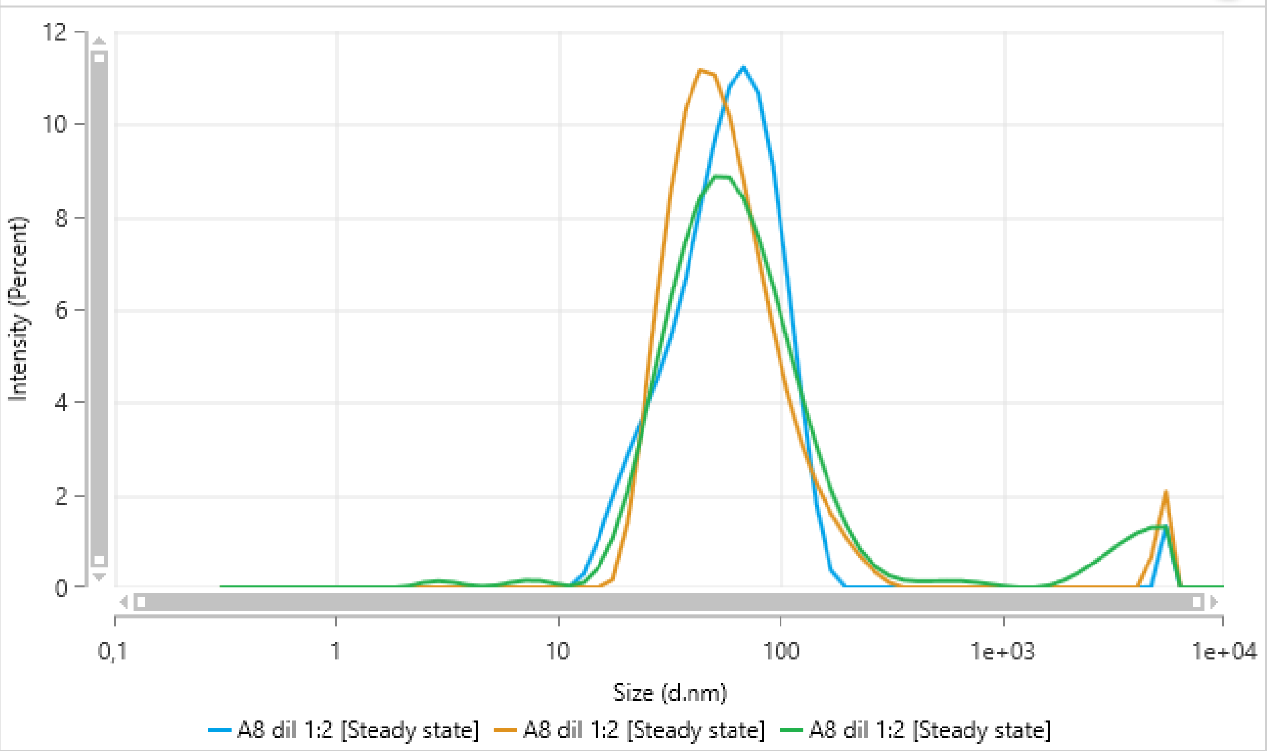

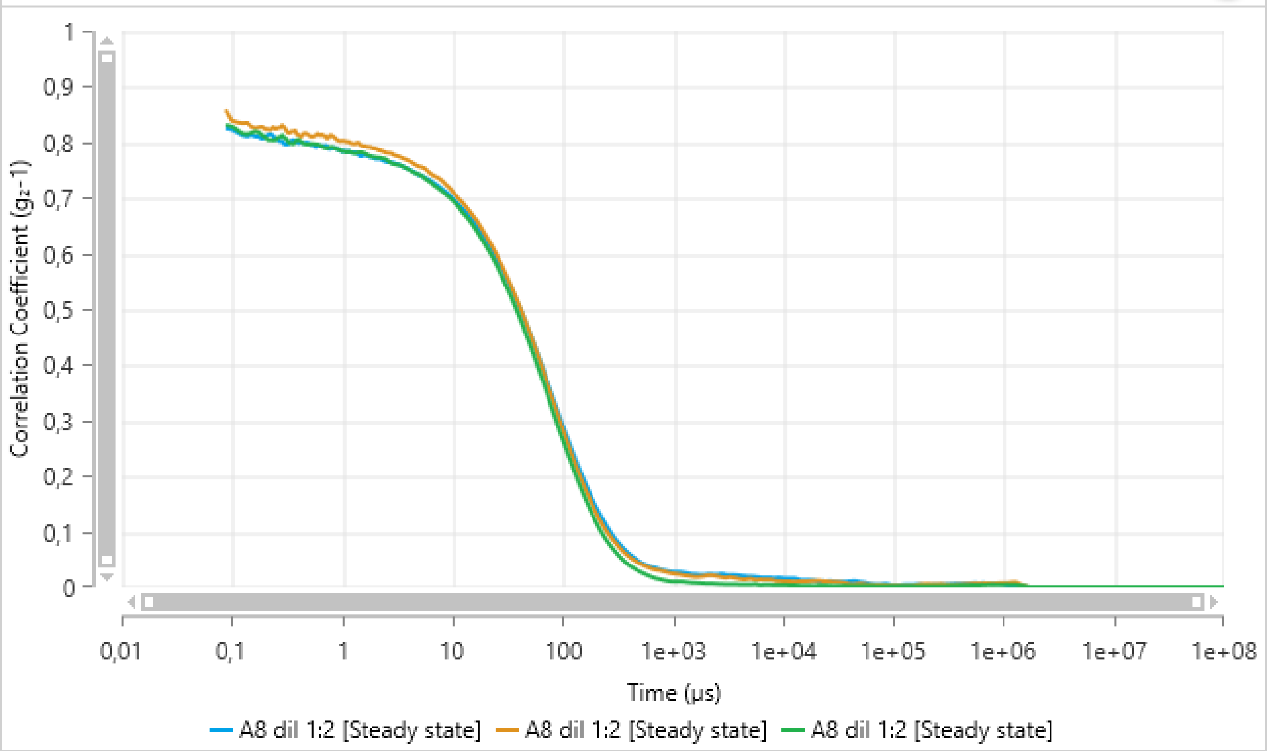

Statistics Table

Name	Mean	Standard Deviation	RSD	Minimum	Maximum
Z-Average (nm)	58,94	4,218	7,157	55,01	63,4
Polydispersity Index (PI)	0,3498	0,02214	6,331	0,3242	0,3639
Peak 1 Mean by Intensity ordered by area (nm)	66,18	4,437	6,704	63,34	71,3
Peak 1 Area by Intensity ordered by area (%)	95,88	3,706	3,865	91,68	98,68
Peak 2 Mean by Intensity ordered by area (nm)	4907	812,9	16,57	3974	5462
Peak 2 Area by Intensity ordered by area (%)	3,511	2,683	76,4	1,322	6,504

LS03

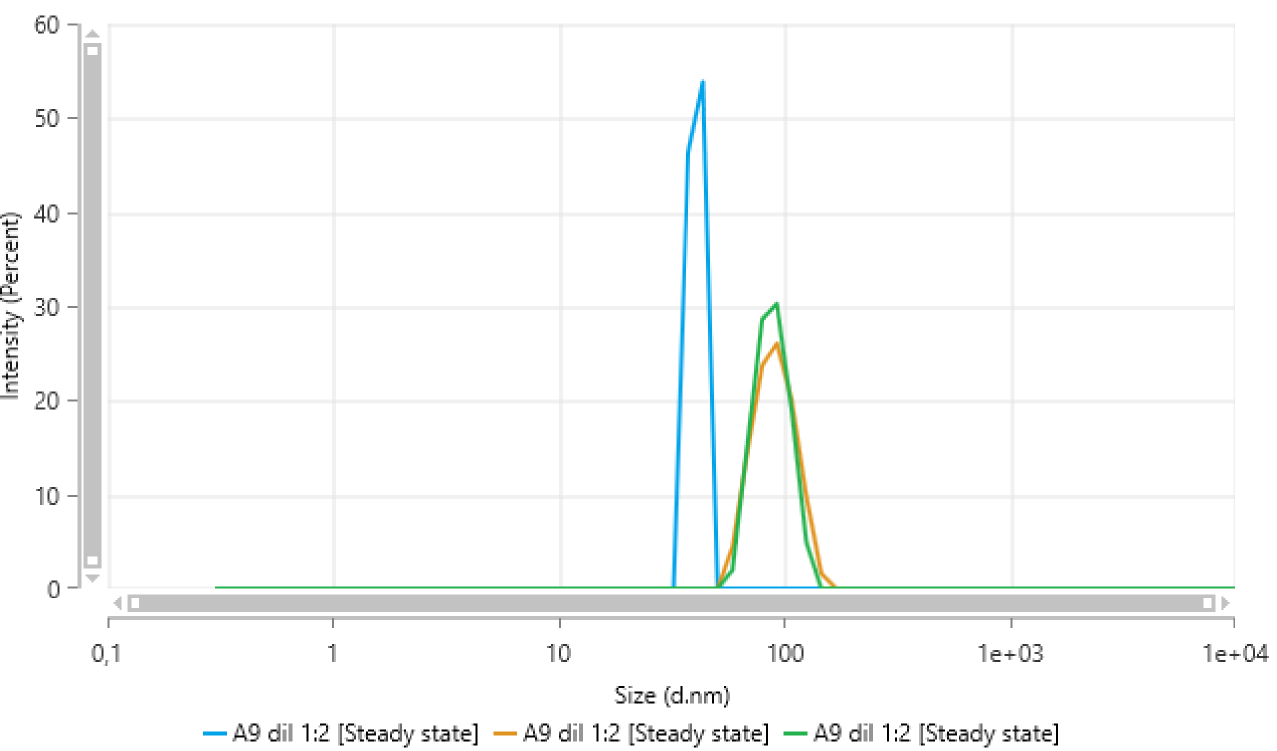

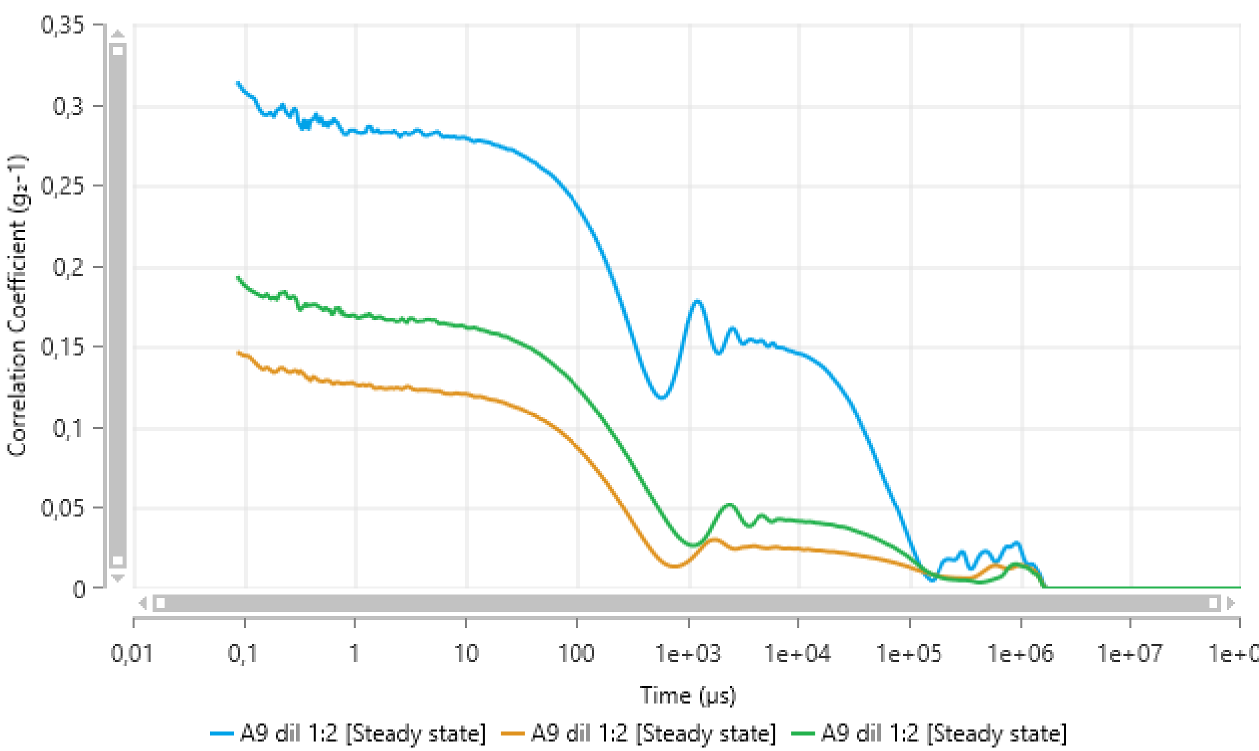

Statistics Table

Name	Mean	Standard Deviation	RSD	Minimum	Maximum
Z-Average (nm)	1039	992,3	95,5	404,7	2183
Polydispersity Index (PI)	0,5956	0,3539	59,42	0,3428	1
Peak 1 Mean by Intensity ordered by area (nm)	74,02	28,75	38,85	40,85	91,91
Peak 1 Area by Intensity ordered by area (%)	100	0	0	100	100

LS06

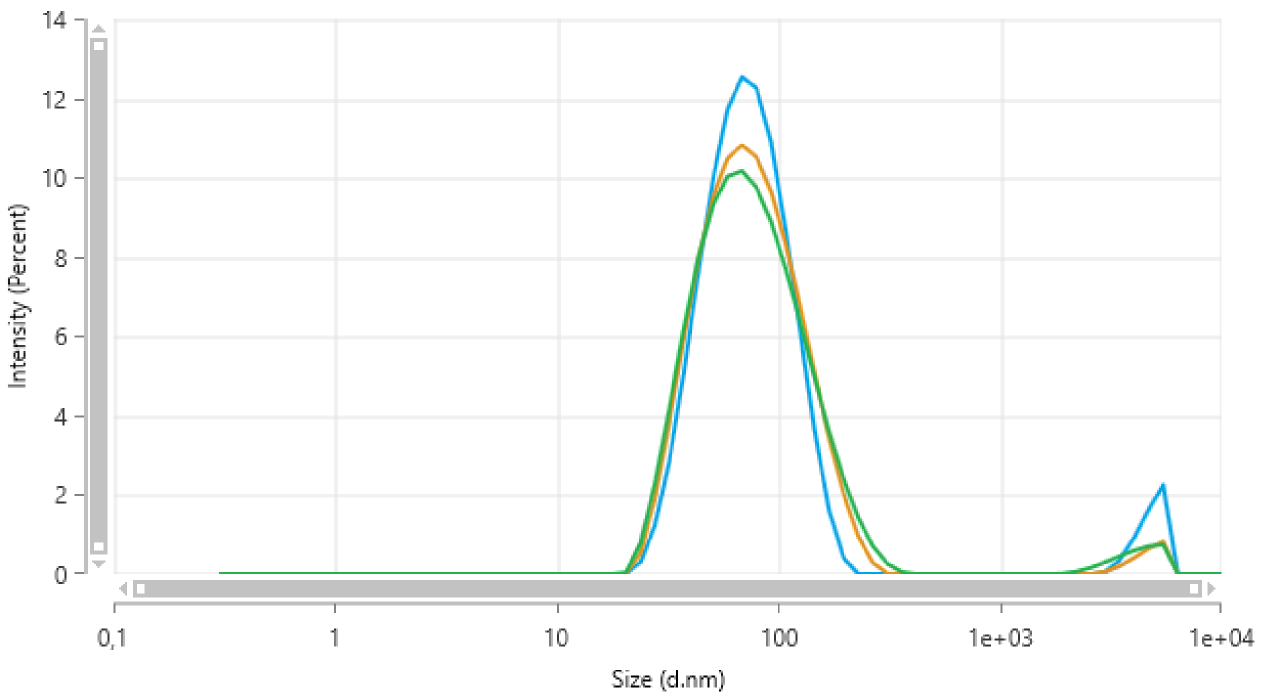

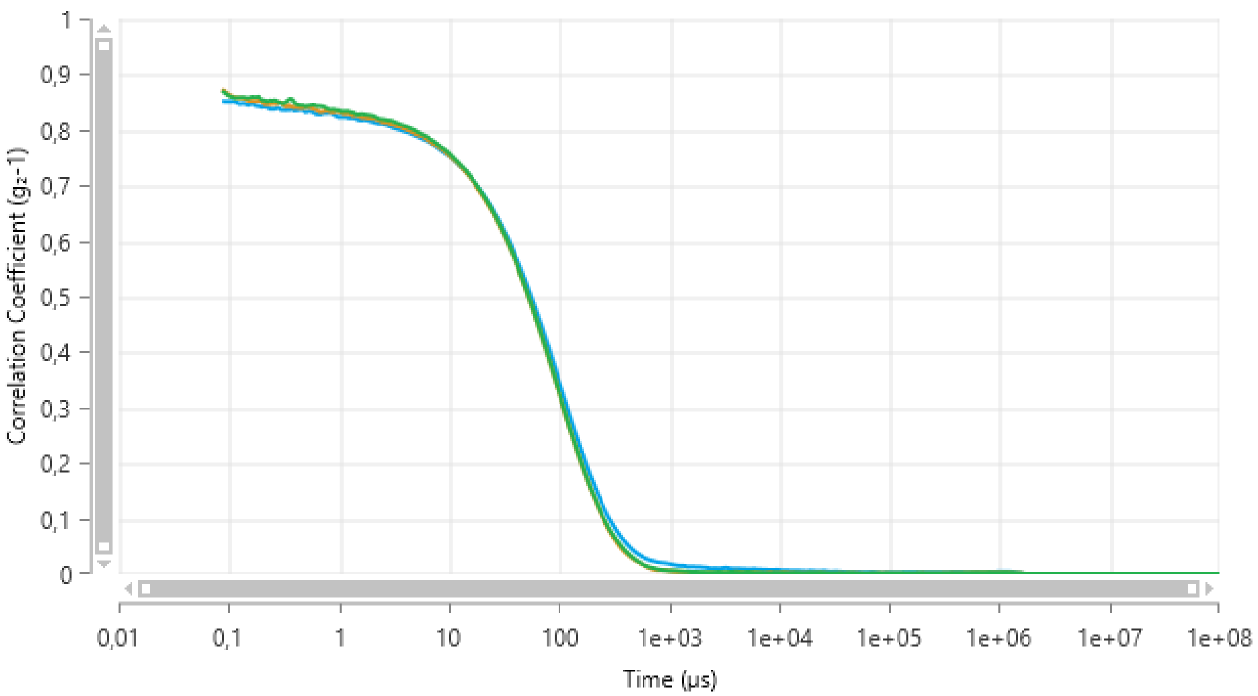

Statistics Table

Name	Mean	Standard Deviation	RSD	Minimum	Maximum
Z-Average (nm)	69,82	2,567	3,676	68,07	72,76
Polydispersity Index (PI)	0,2685	0,0425	15,83	0,2371	0,3169
Peak 1 Mean by Intensity ordered by area (nm)	81,44	4,075	5,004	76,87	84,69
Peak 1 Area by Intensity ordered by area (%)	96,53	1,578	1,635	94,79	97,85
Peak 2 Mean by Intensity ordered by area (nm)	4588	309,9	6,756	4235	4817
Peak 2 Area by Intensity ordered by area (%)	3,465	1,578	45,54	2,147	5,214

LS06

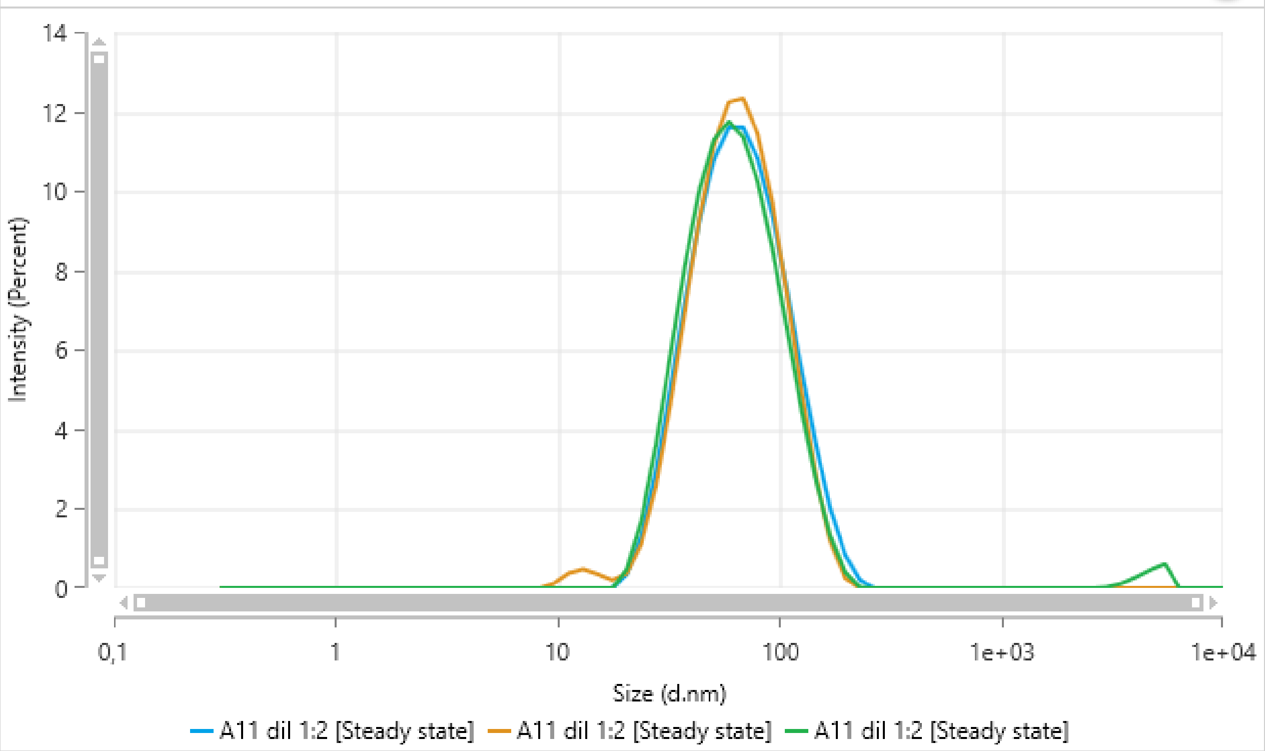

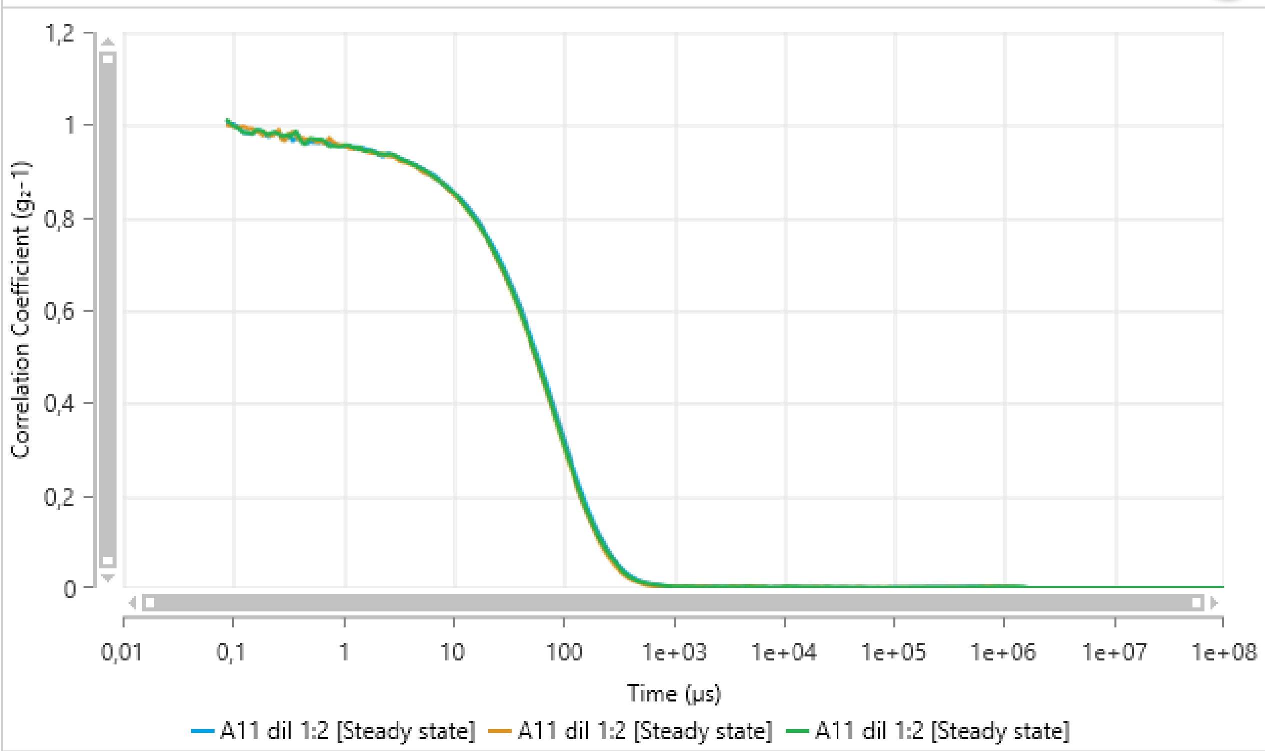

Statistics Table

Name	Mean	Standard Deviation	RSD	Minimum	Maximum
Z-Average (nm)	57,7	1,313	2,275	56,85	59,21
Polydispersity Index (PI)	0,2037	0,01623	7,965	0,1853	0,216
Peak 1 Mean by Intensity ordered by area (nm)	70,63	2,439	3,454	68,22	73,1
Peak 1 Area by Intensity ordered by area (%)	99	0,8671	0,8758	98,48	100
Peak 2 Mean by Intensity ordered by area (nm)	2390	3362	140,6	13,43	4767
Peak 2 Area by Intensity ordered by area (%)	1,501	0,03004	2,001	1,48	1,523

LS06

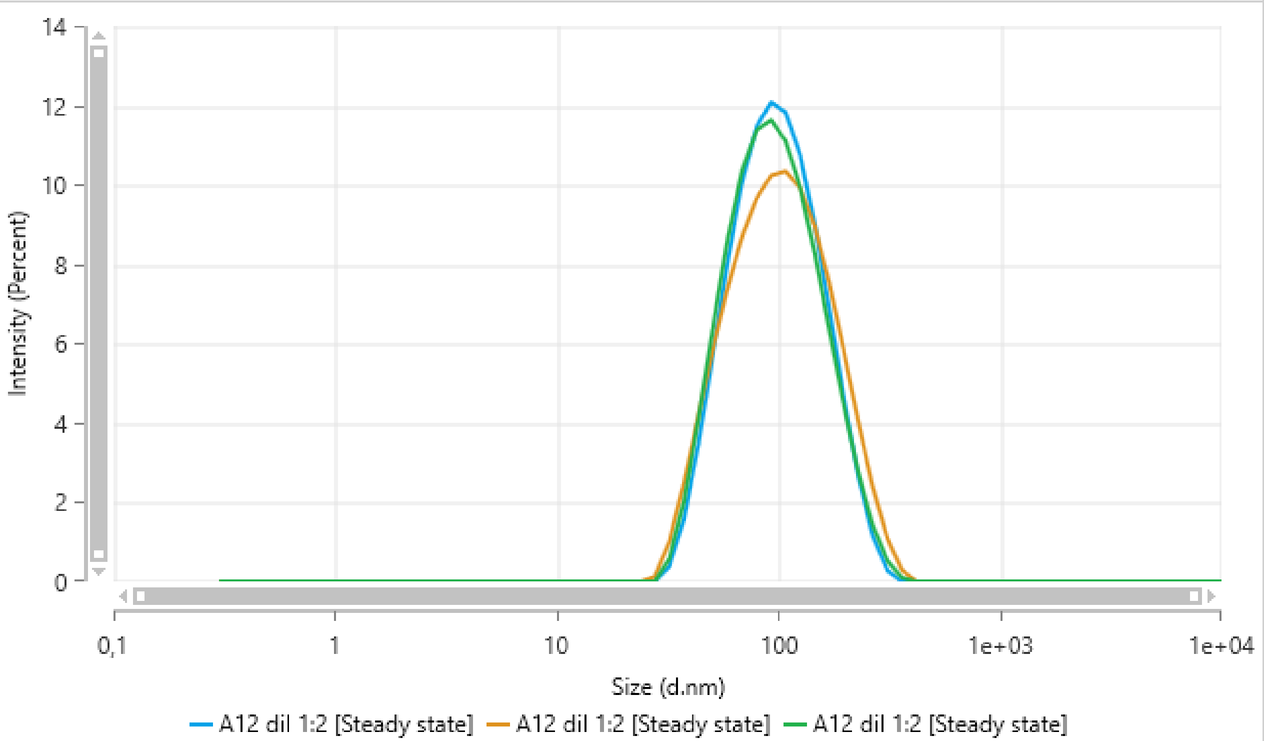

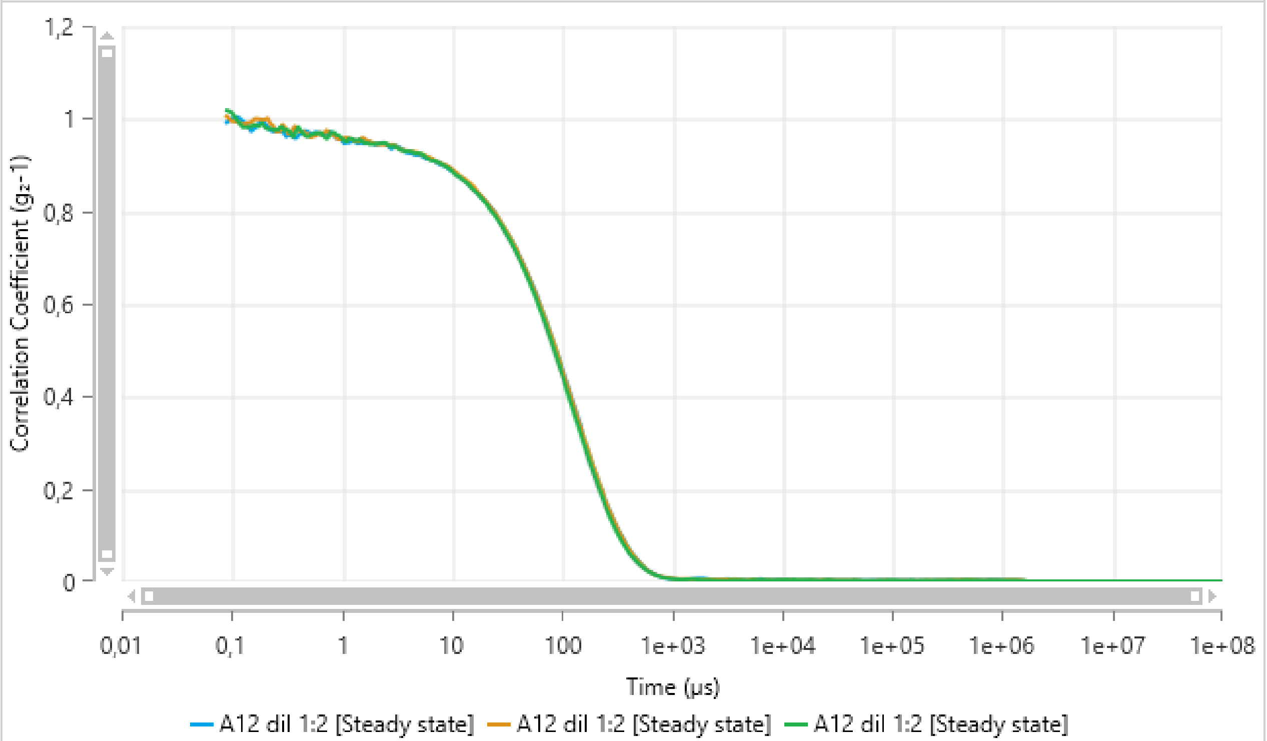

Statistics Table

Name	Mean	Standard Deviation	RSD	Minimum	Maximum
Z-Average (nm)	88,43	1,964	2,221	86,19	89,87
Polydispersity Index (PI)	0,1965	0,008902	4,53	0,1886	0,2061
Peak 1 Mean by Intensity ordered by area (nm)	109,6	4,554	4,156	106,4	114,8
Peak 1 Area by Intensity ordered by area (%)	100	0	0	100	100

LS07

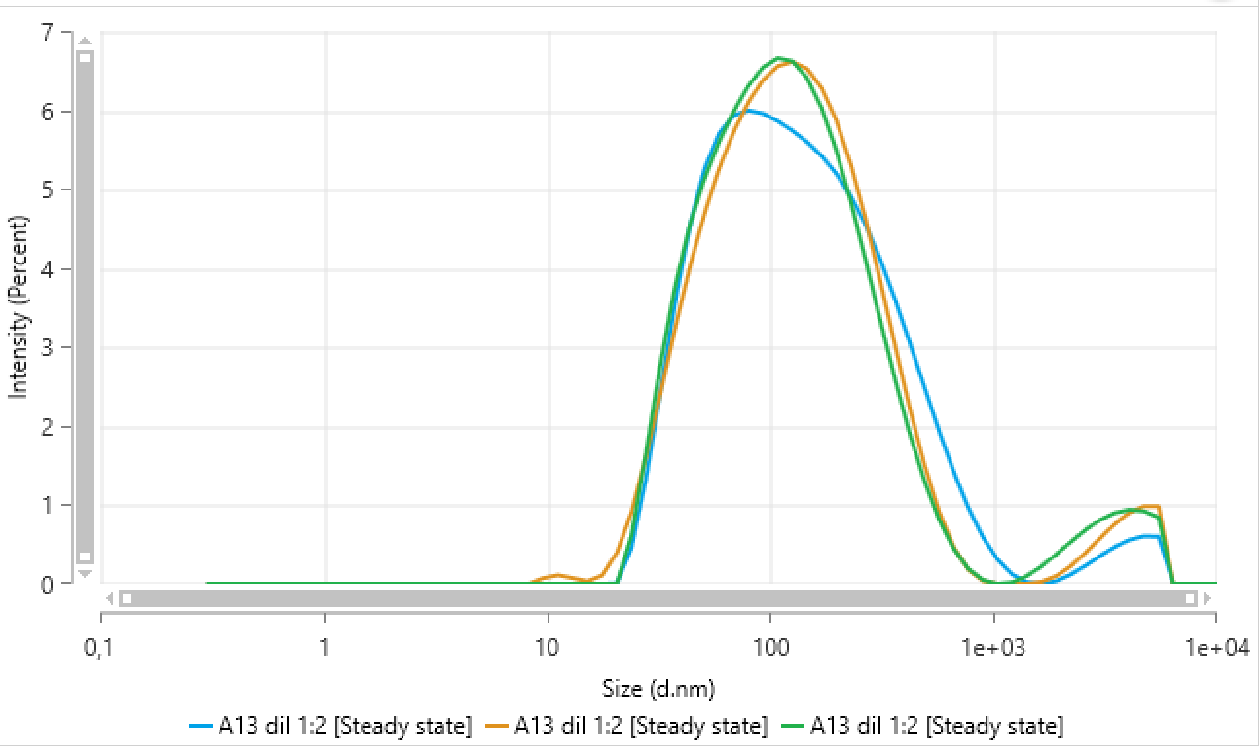

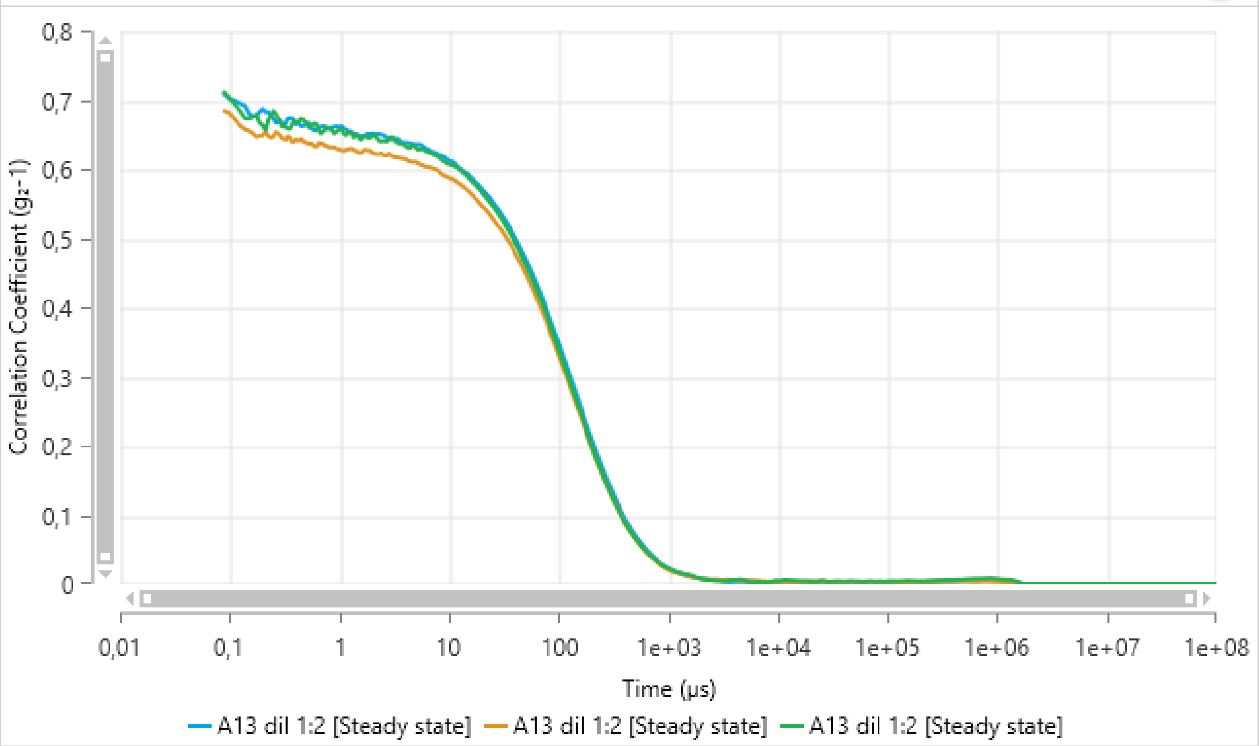

Statistics Table

Name	Mean	Standard Deviation	RSD	Minimum	Maximum
Z-Average (nm)	98,76	1,802	1,824	97,07	100,7
Polydispersity Index (PI)	0,4161	0,01091	2,621	0,4039	0,4248
Peak 1 Mean by Intensity ordered by area (nm)	161,7	19,64	12,15	147	184
Peak 1 Area by Intensity ordered by area (%)	95,16	1,716	1,804	93,67	97,04
Peak 2 Mean by Intensity ordered by area (nm)	3855	274,1	7,109	3540	4035
Peak 2 Area by Intensity ordered by area (%)	4,744	1,692	35,66	2,963	6,329

LS07

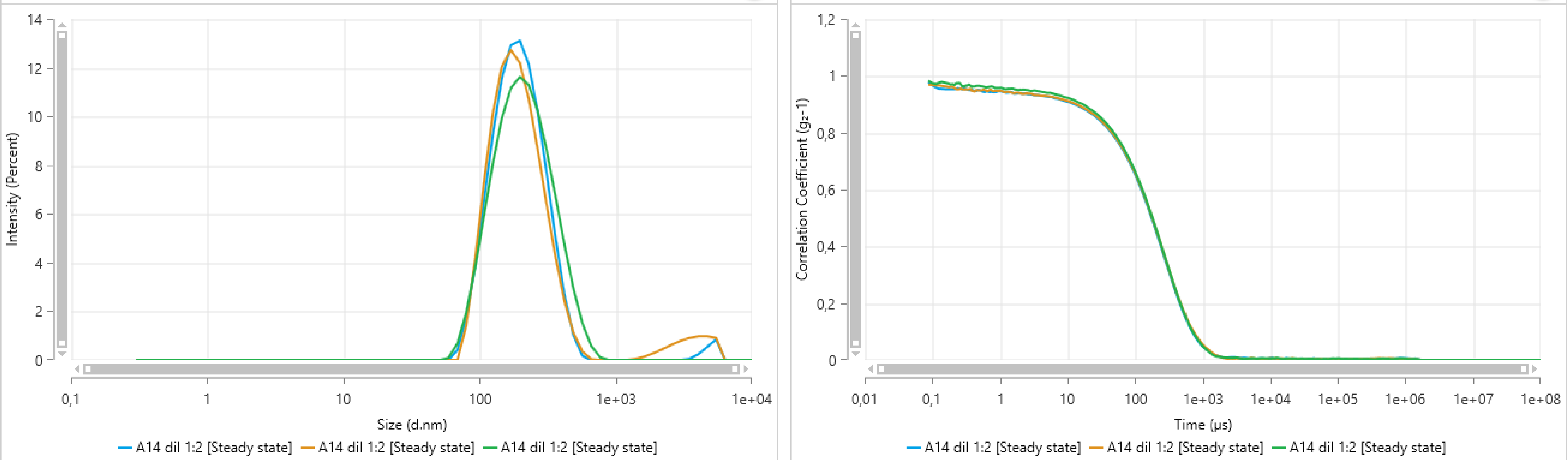

Statistics Table

Name	Mean	Standard Deviation	RSD	Minimum	Maximum
Z-Average (nm)	183,8	0,9651	0,525	182,9	184,8
Polydispersity Index (PI)	0,2118	0,01621	7,651	0,2004	0,2303
Peak 1 Mean by Intensity ordered by area (nm)	213,1	15,37	7,215	202	230,6
Peak 1 Area by Intensity ordered by area (%)	97,42	3,151	3,234	93,91	100
Peak 2 Mean by Intensity ordered by area (nm)	4320	909,6	21,06	3677	4963
Peak 2 Area by Intensity ordered by area (%)	3,876	3,137	80,94	1,657	6,094

LS07

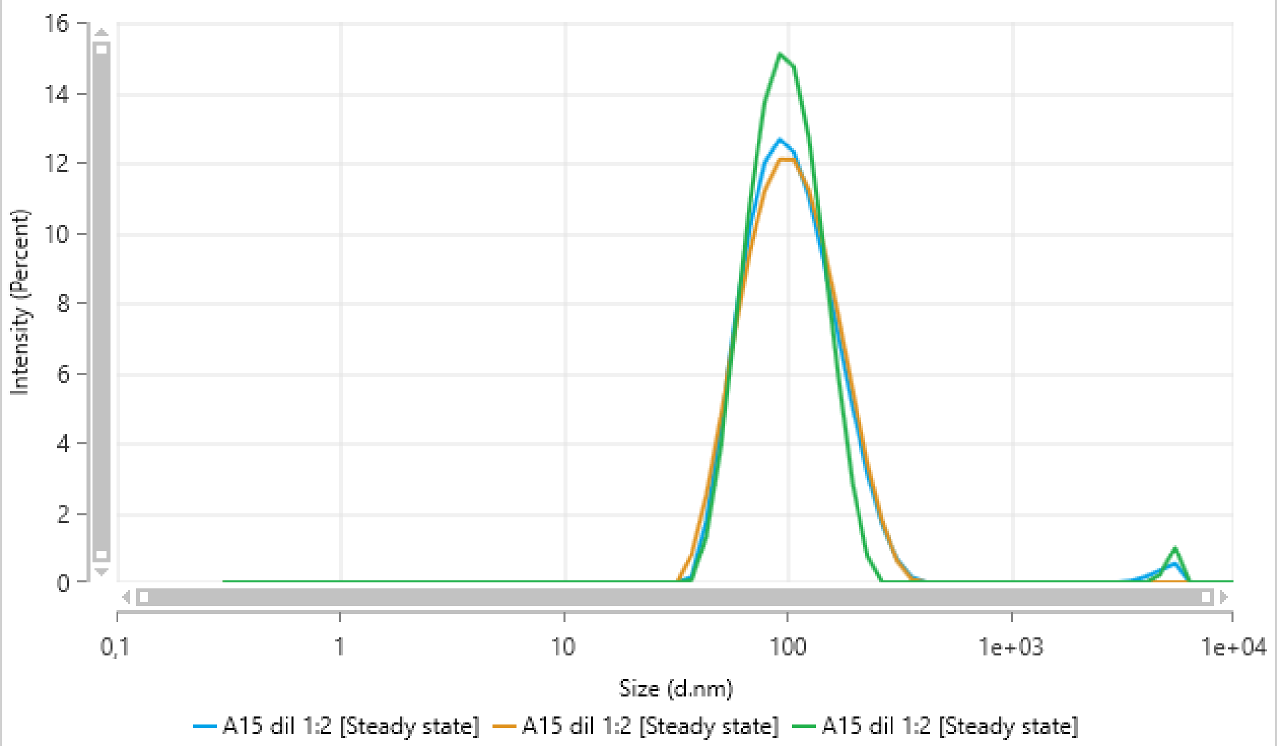

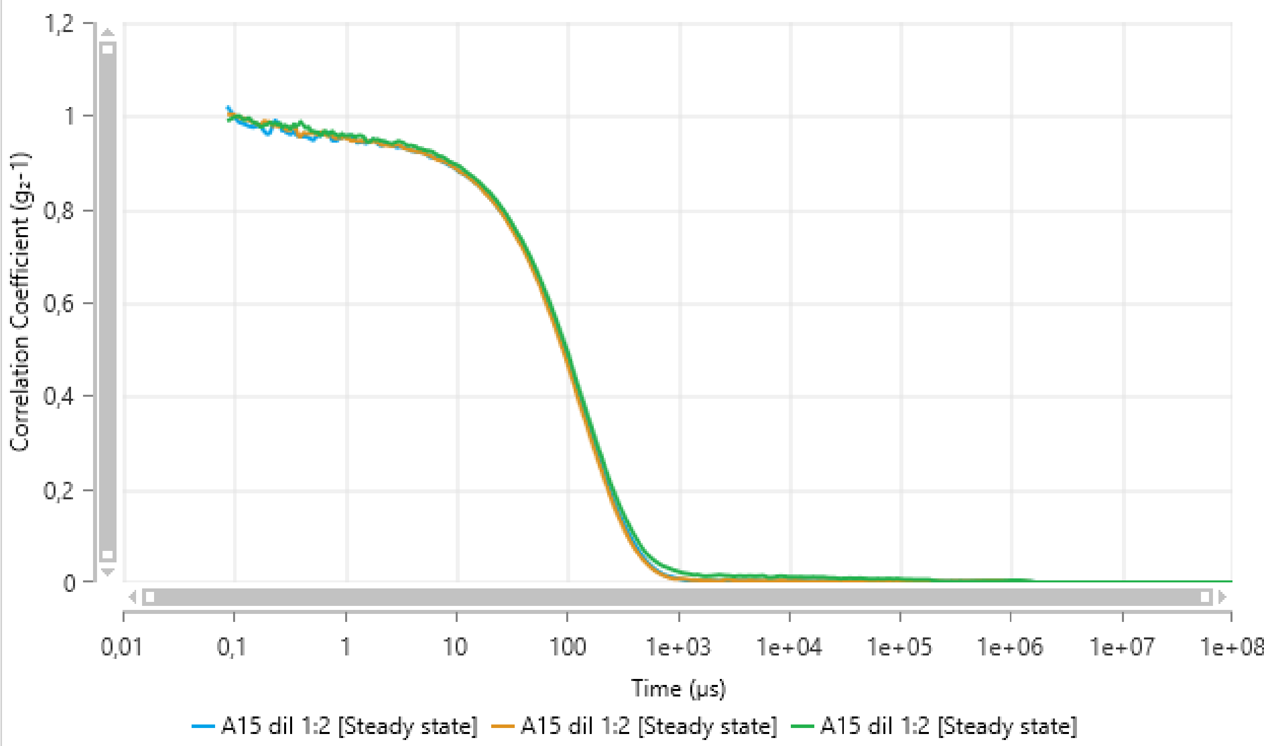

Statistics Table

Name	Mean	Standard Deviation	RSD	Minimum	Maximum
Z-Average (nm)	97,14	2,221	2,287	94,87	99,31
Polydispersity Index (PI)	0,2024	0,04249	20,99	0,1667	0,2494
Peak 1 Mean by Intensity ordered by area (nm)	110,6	6,013	5,435	103,7	114,5
Peak 1 Area by Intensity ordered by area (%)	99,23	0,6699	0,675	98,78	100
Peak 2 Mean by Intensity ordered by area (nm)	5128	277,9	5,419	4931	5324
Peak 2 Area by Intensity ordered by area (%)	1,154	0,1006	8,719	1,083	1,225

Z37

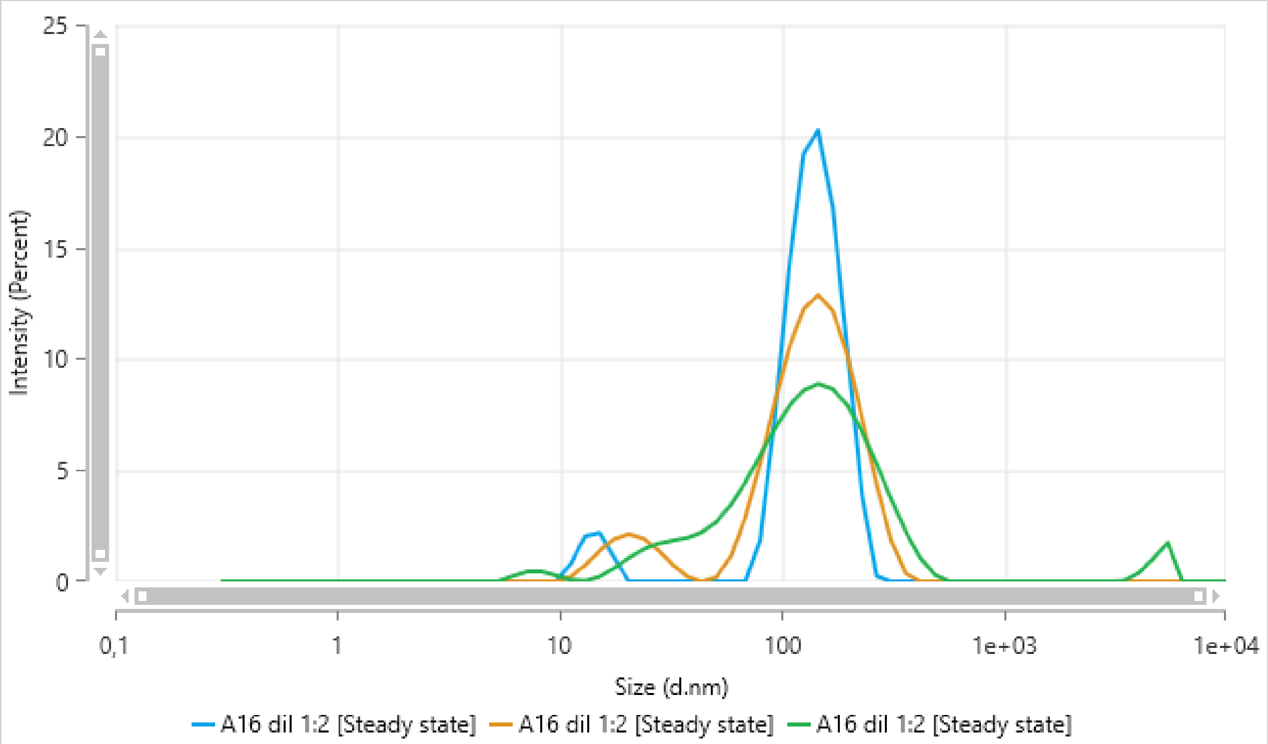

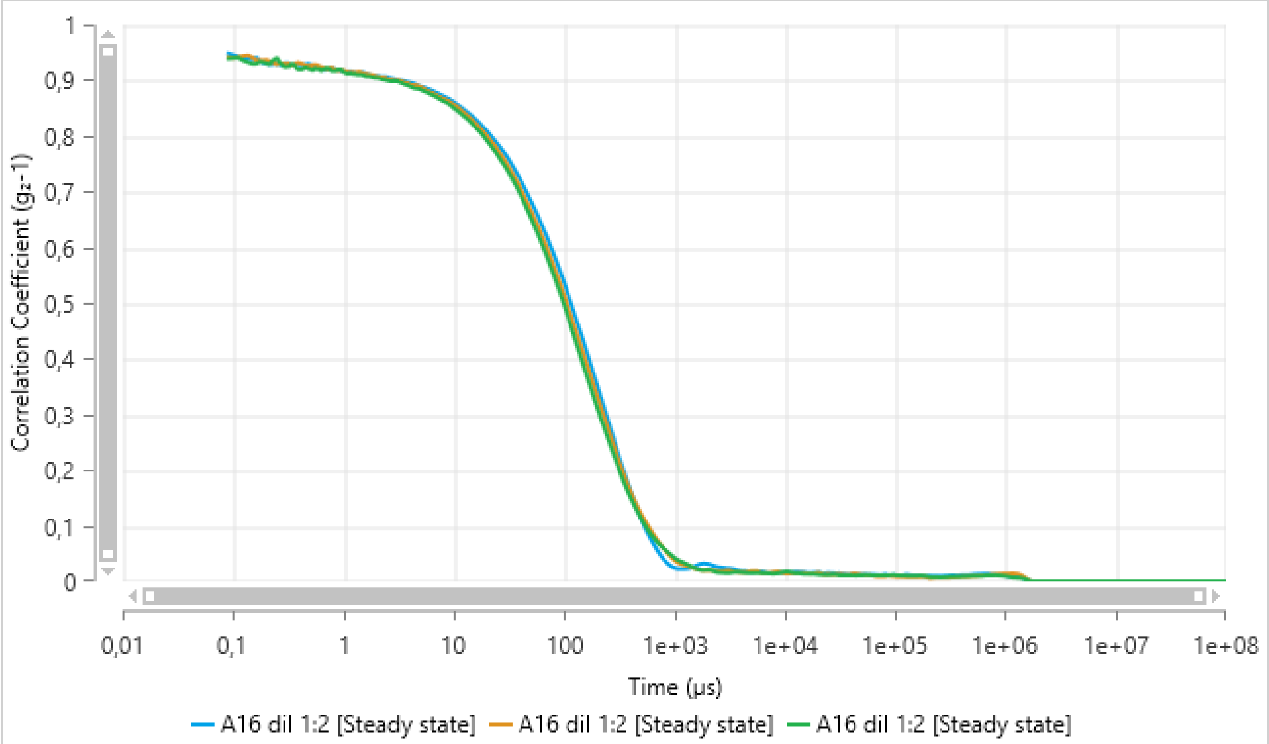

Statistics Table

Name	Mean	Standard Deviation	RSD	Minimum	Maximum
Z-Average (nm)	114,9	9,946	8,658	104,7	124,6
Polydispersity Index (PI)	0,3751	0,05773	15,39	0,3156	0,4309
Peak 1 Mean by Intensity ordered by area (nm)	147,5	4,433	3,005	144,6	152,6
Peak 1 Area by Intensity ordered by area (%)	92,85	3,133	3,374	89,32	95,29
Peak 2 Mean by Intensity ordered by area (nm)	1687	2891	171,4	14,39	5026
Peak 2 Area by Intensity ordered by area (%)	6,646	3,778	56,85	3,195	10,68

Z37

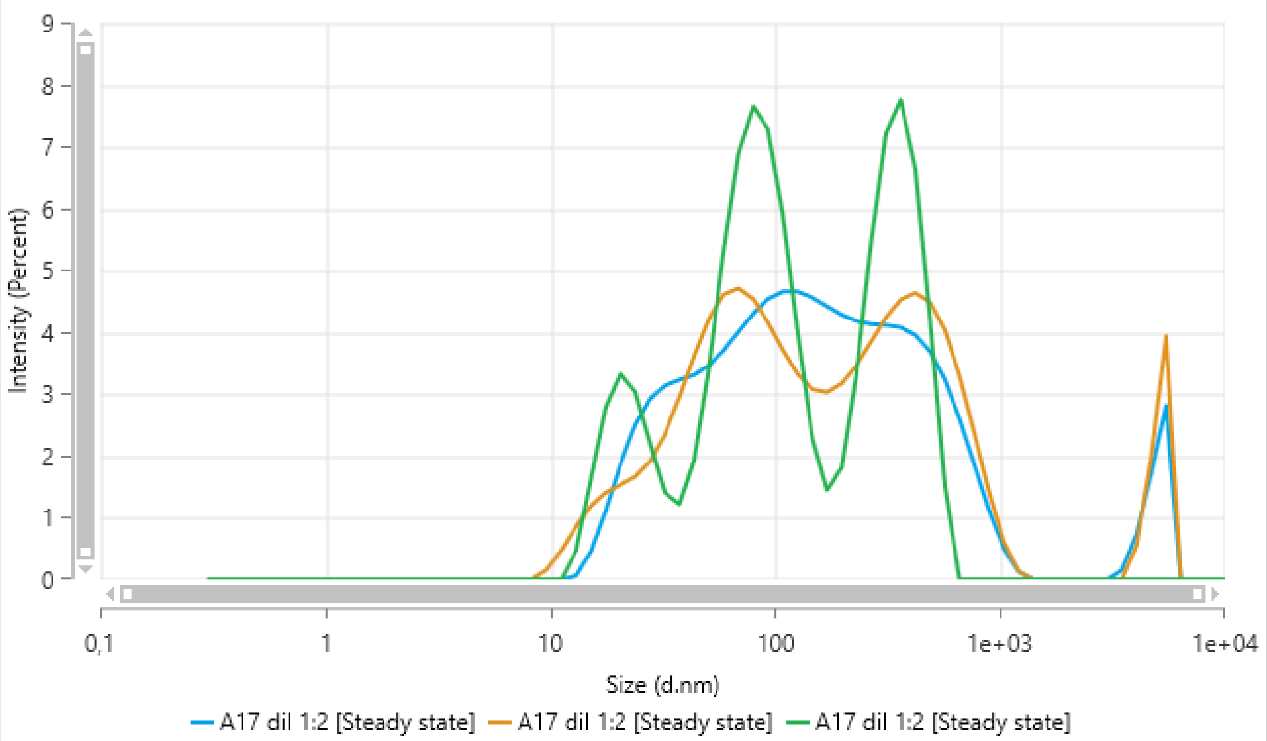

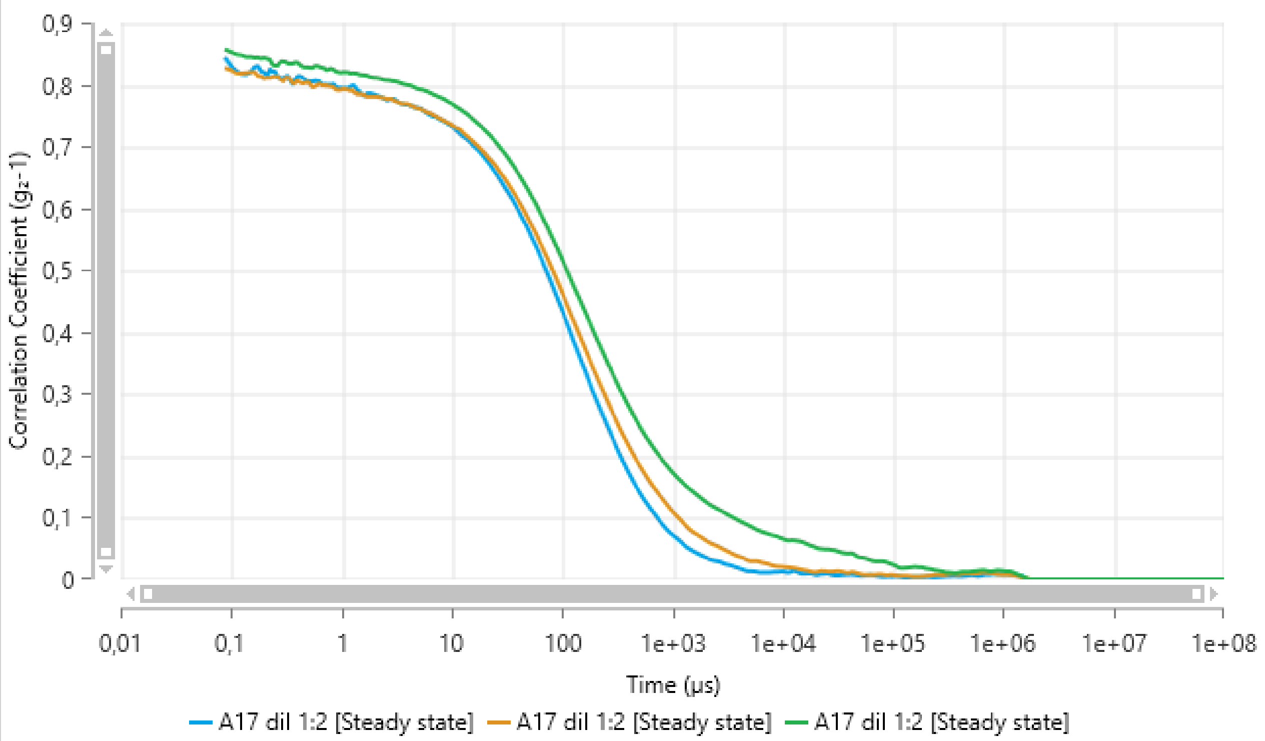

Statistics Table

Name	Mean	Standard Deviation	RSD	Minimum	Maximum
Z-Average (nm)	165,8	59,74	36,03	114	231,2
Polydispersity Index (PI)	0,474	0,04381	9,242	0,4327	0,52
Peak 1 Mean by Intensity ordered by area (nm)	124,4	78,01	62,73	72,39	214,1
Peak 1 Area by Intensity ordered by area (%)	64,19	26,49	41,26	46,13	94,6
Peak 2 Mean by Intensity ordered by area (nm)	1916	2651	138,4	344,7	4977
Peak 2 Area by Intensity ordered by area (%)	28,52	20,1	70,49	5,402	41,89

Z37

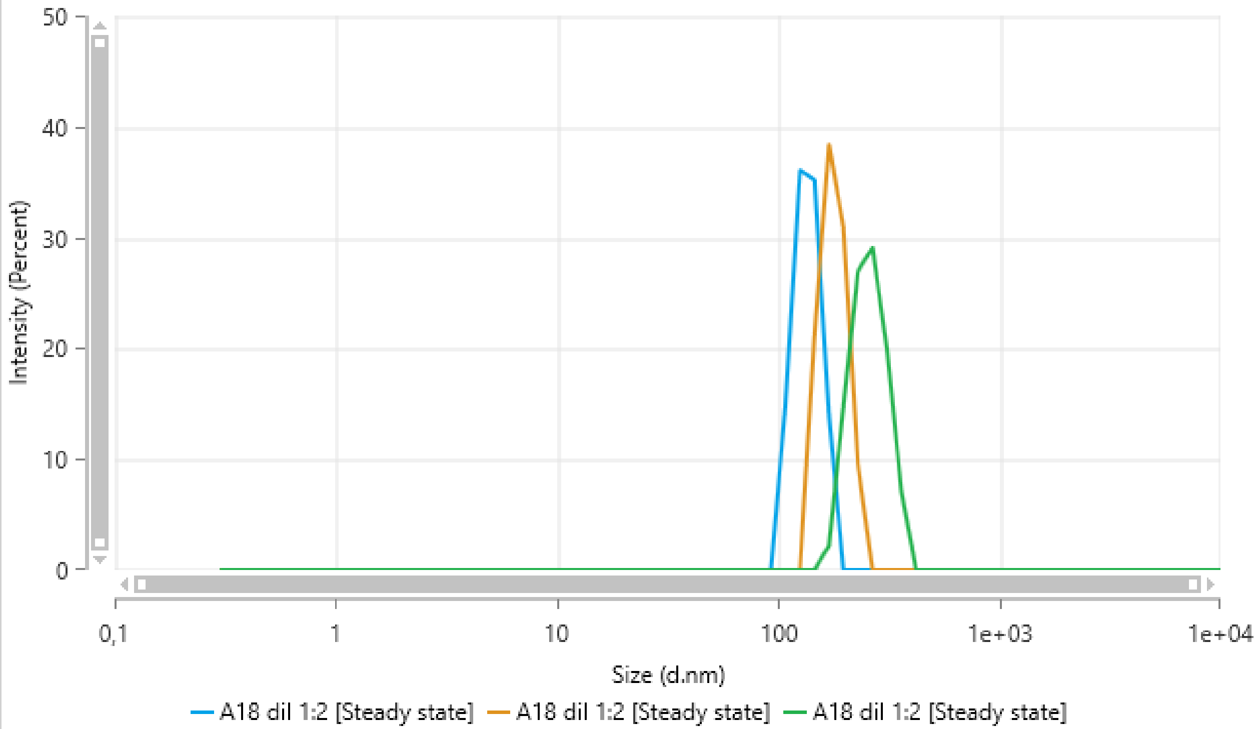

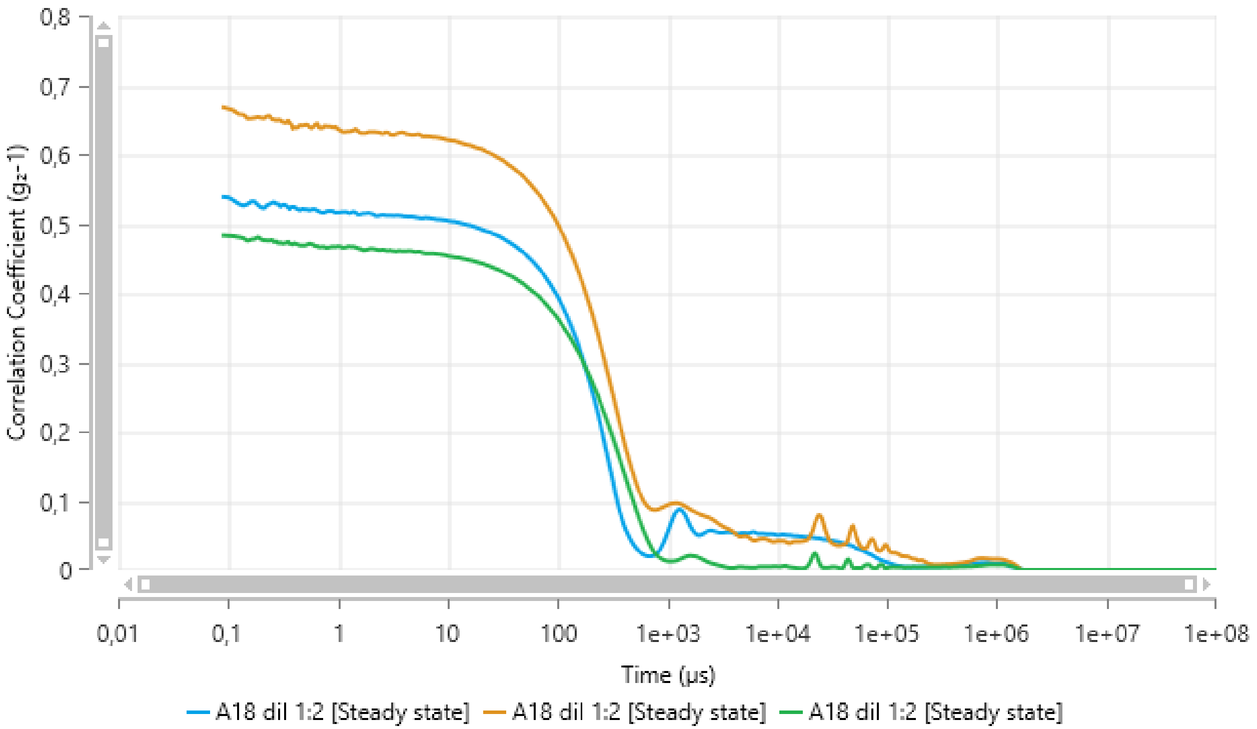

Statistics Table

Name	Mean	Standard Deviation	RSD	Minimum	Maximum
Z-Average (nm)	263,4	23,96	9,096	238,5	286,2
Polydispersity Index (PI)	0,2336	0,09916	42,45	0,1192	0,2959
Peak 1 Mean by Intensity ordered by area (nm)	191,9	62,85	32,75	136,4	260,1
Peak 1 Area by Intensity ordered by area (%)	100	0	0	100	100
