# Supplementary material for: Establishment and application of a vesicle extraction method for clinical strains of Pseudomonas aeruginosa
Source: Microbiol Spectr. 2026 May 12;14(6):e03746-25. doi: 10.1128/spectrum.03746-25 (PMC13227995; doi:10.1128/spectrum.03746-25)
Supplement: Supplemental figures — Fig. S1 to S5. [file spectrum.03746-25-s0003.docx]

Supplemental material

Methods and Protocols article

Establishment and application of a vesicle extraction method for clinical strains of *Pseudomonas aeruginosa*

Tania Henriquez^1#^, Francesco Santoro^1^, Lorenzo Colombini^1^, Donata Medaglini^1^, Lucia Pallecchi^1^, Saioa Sanz^1^, Ilaria Clemente^2^, Agnese Magnani^2^, Eugenio Paccagnini^3^, Mariangela Gentile^3^, Pietro Lupetti^3^, Serena Schwenkert^4^, Chiara Falciani^1^

^1^Department of Medical Biotechnologies, University of Siena, Siena 53100, Italy.

^2^Department of Biotechnology, Chemistry and Pharmacy, University of Siena, Siena, Italy.

^3^Department of Life Sciences, University of Siena, Siena, Italy.

^4^Mass Spectrometry of Biomolecules (MSBioLMU), LMU Munich, Großhaderner Str. 2-4, 82152 Planegg-Martinsried, Germany.

^#^Correspondence to Tania Henriquez. Email: tania.henriquez@unisi.it

Keywords: Extracellular vesicles, *Pseudomonas aeruginosa,* Vesicle extraction.


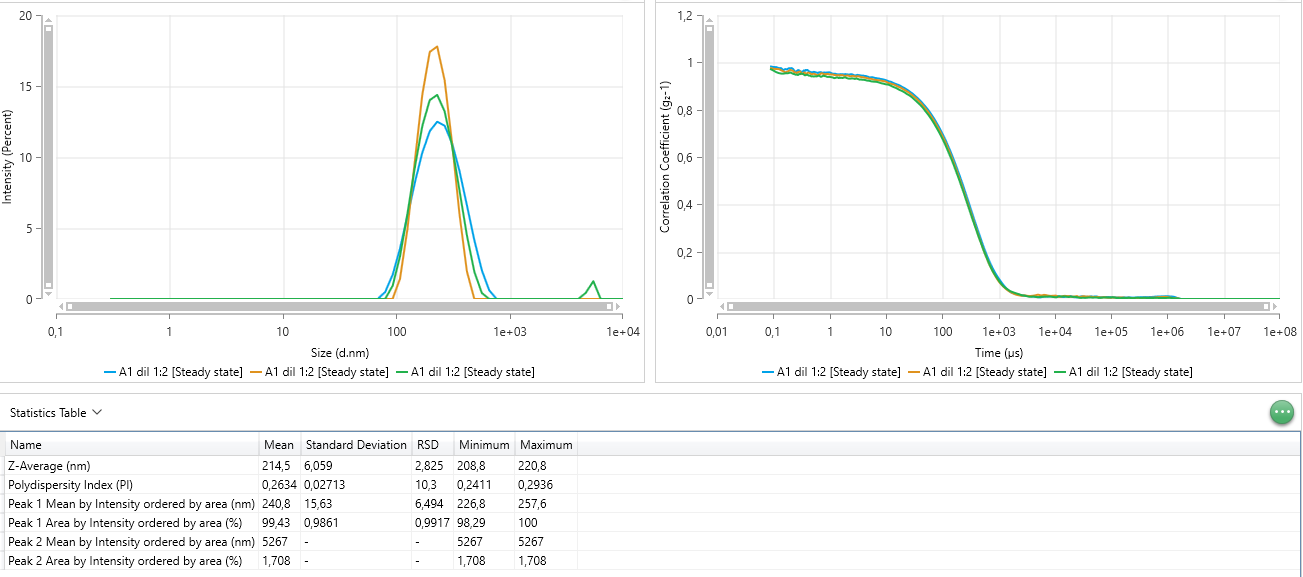


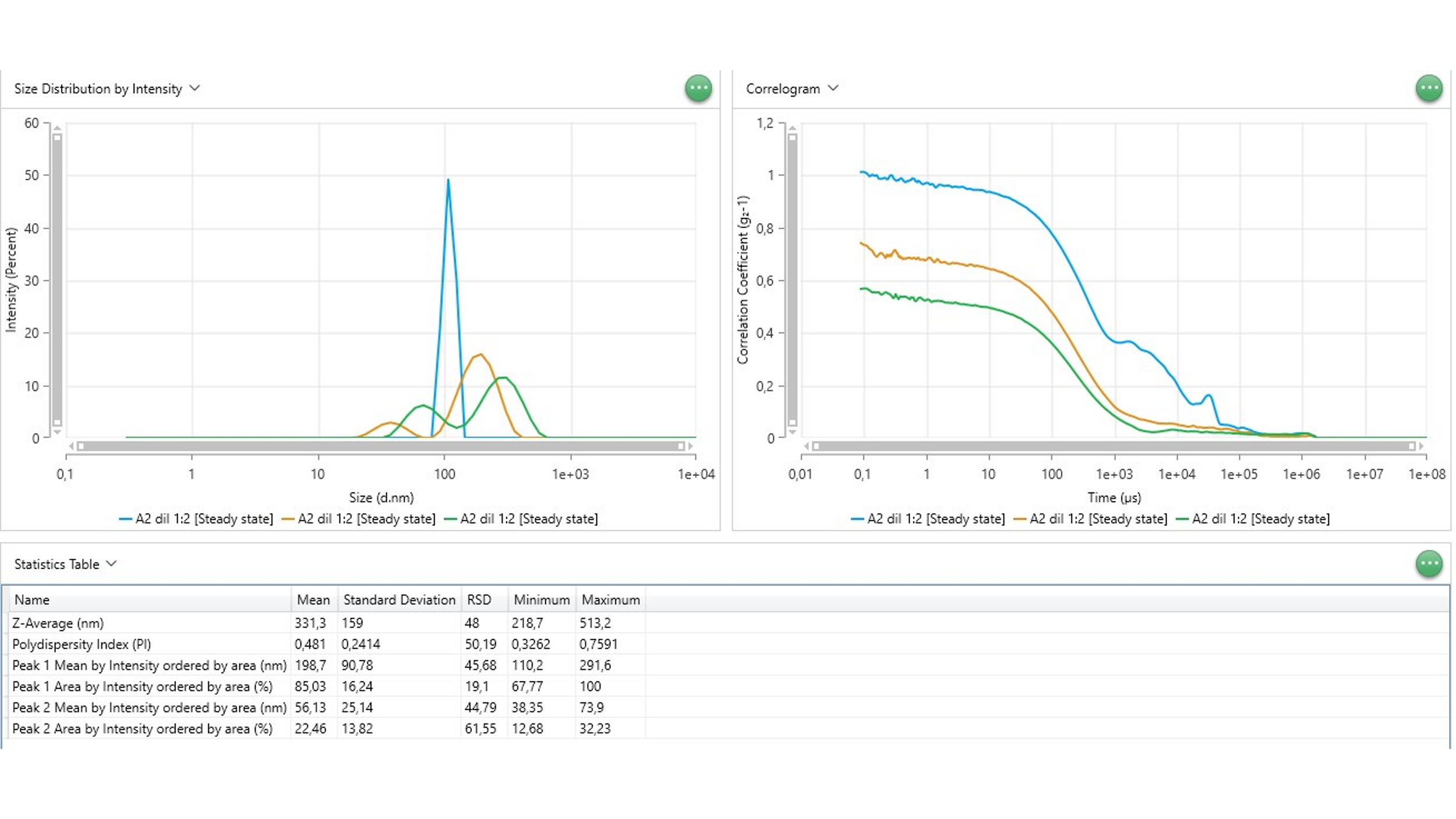


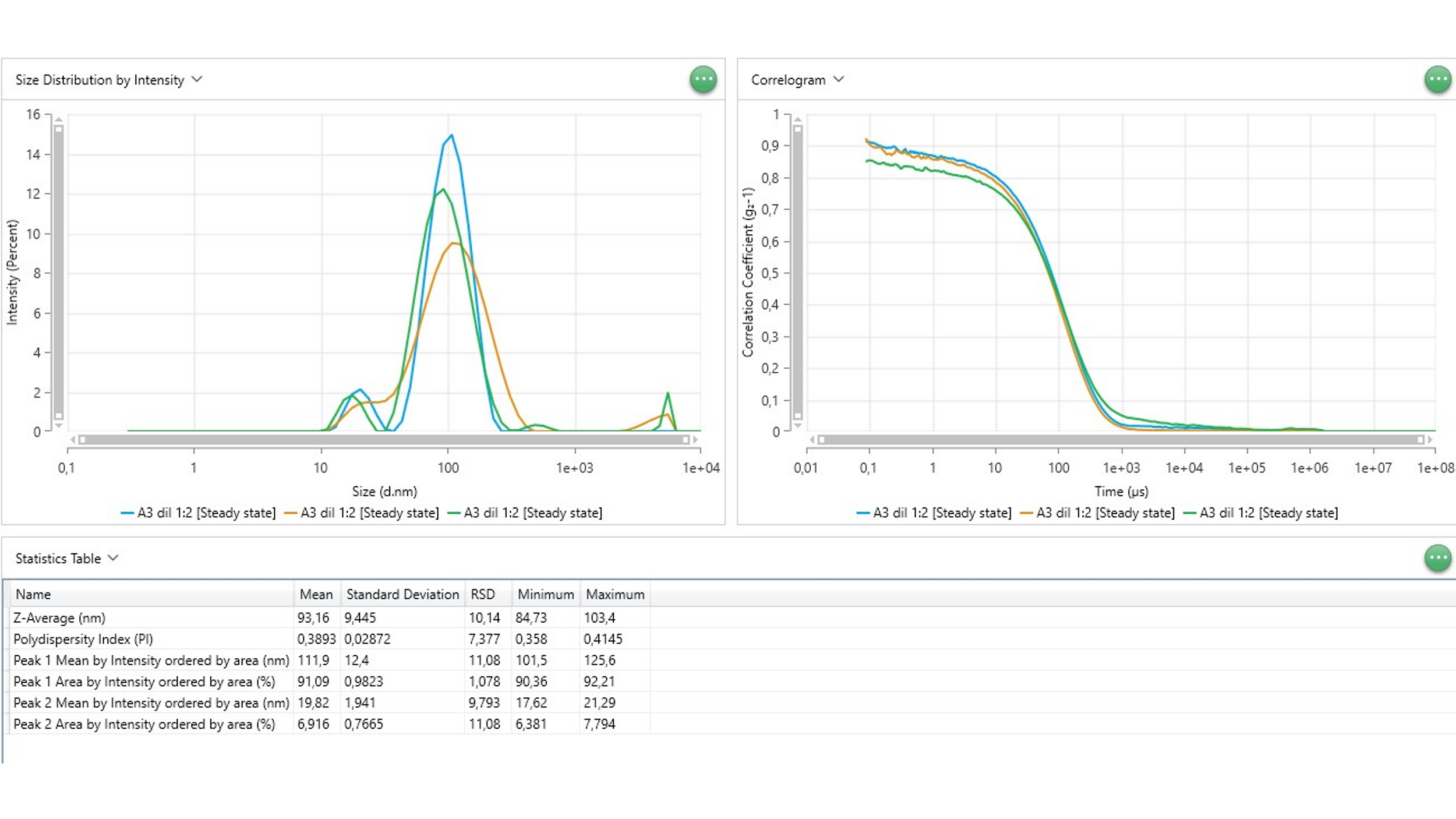


Figure S1. Dynamic light scattering analysis of PAO1 vesicles extracted with our in-house method (results from 3 replicates are shown).


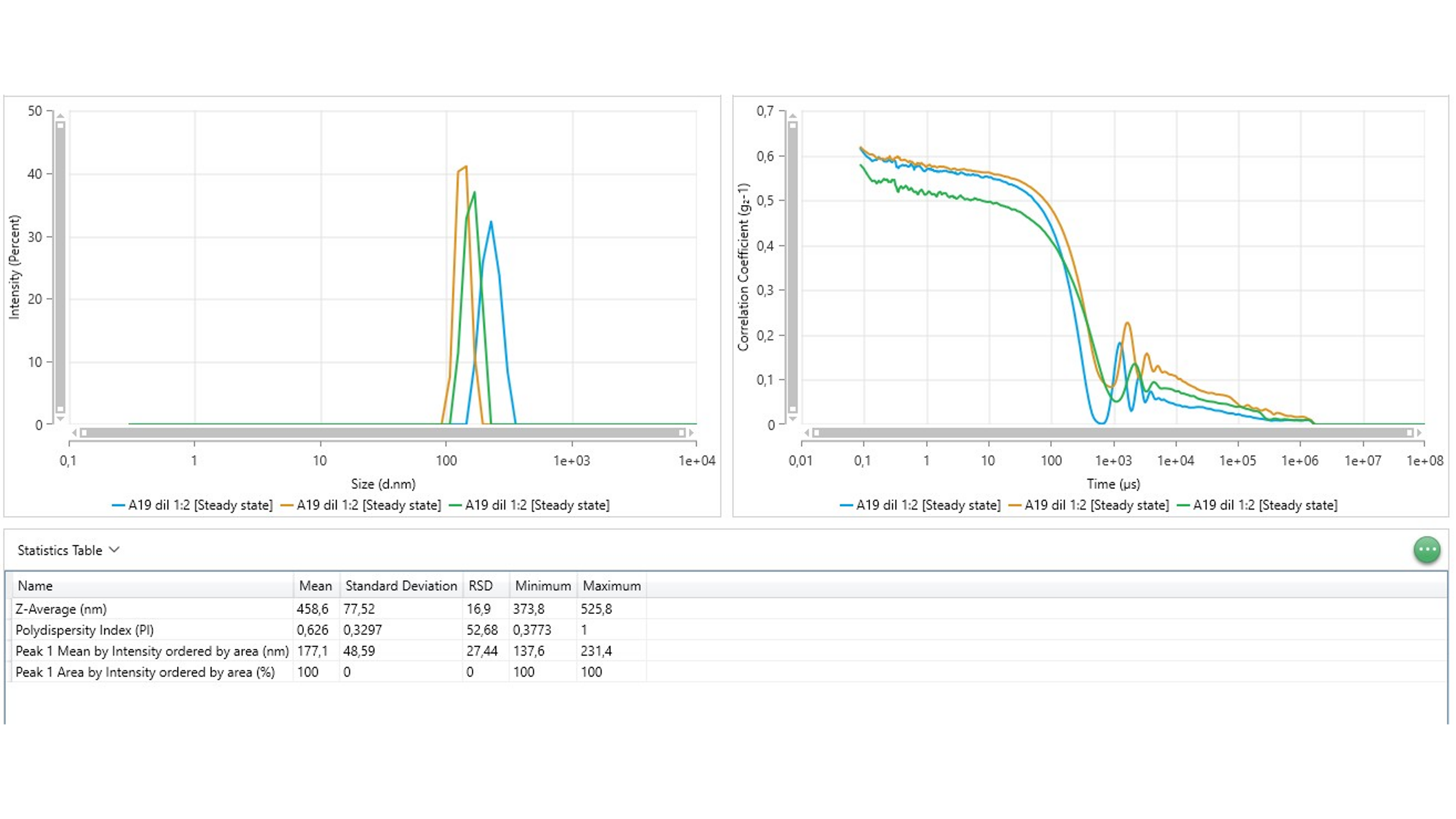


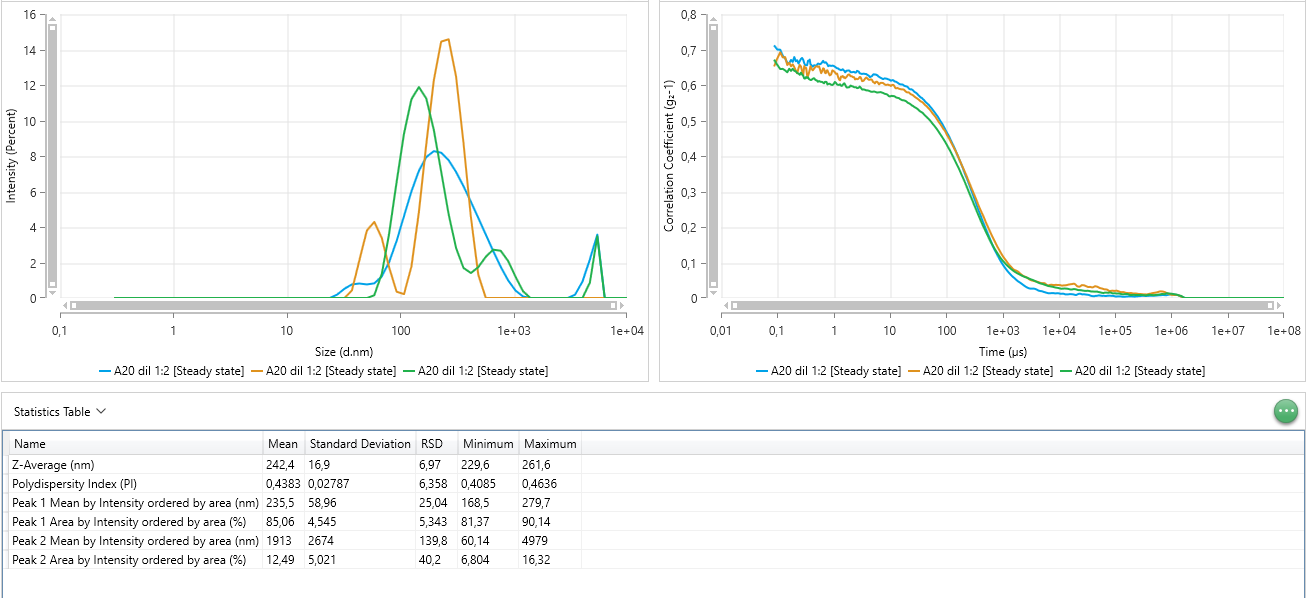


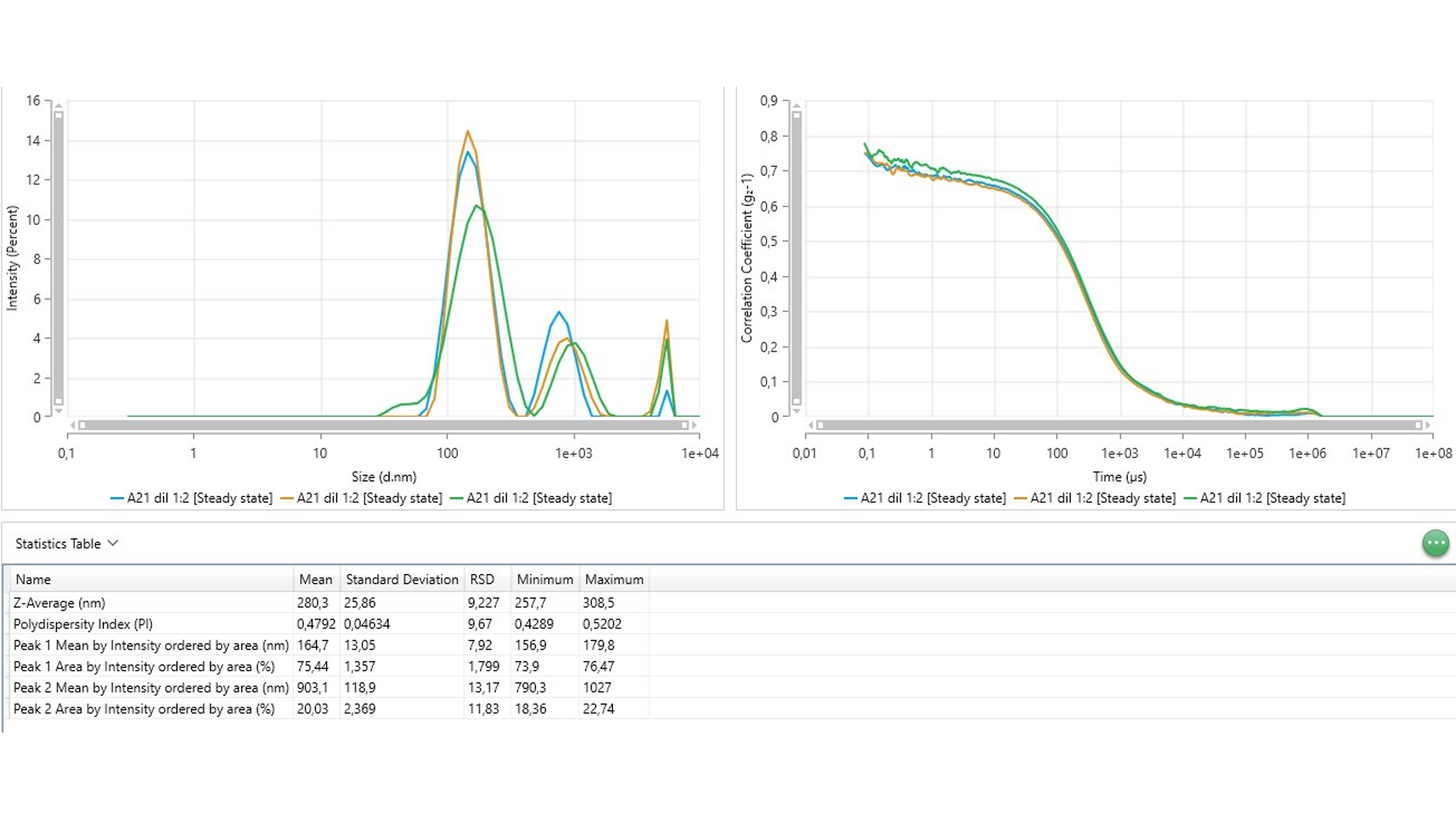


Figure S2. Dynamic light scattering analysis of PAO1 vesicles extracted by ultracentrifugation (results from 3 replicates are shown).


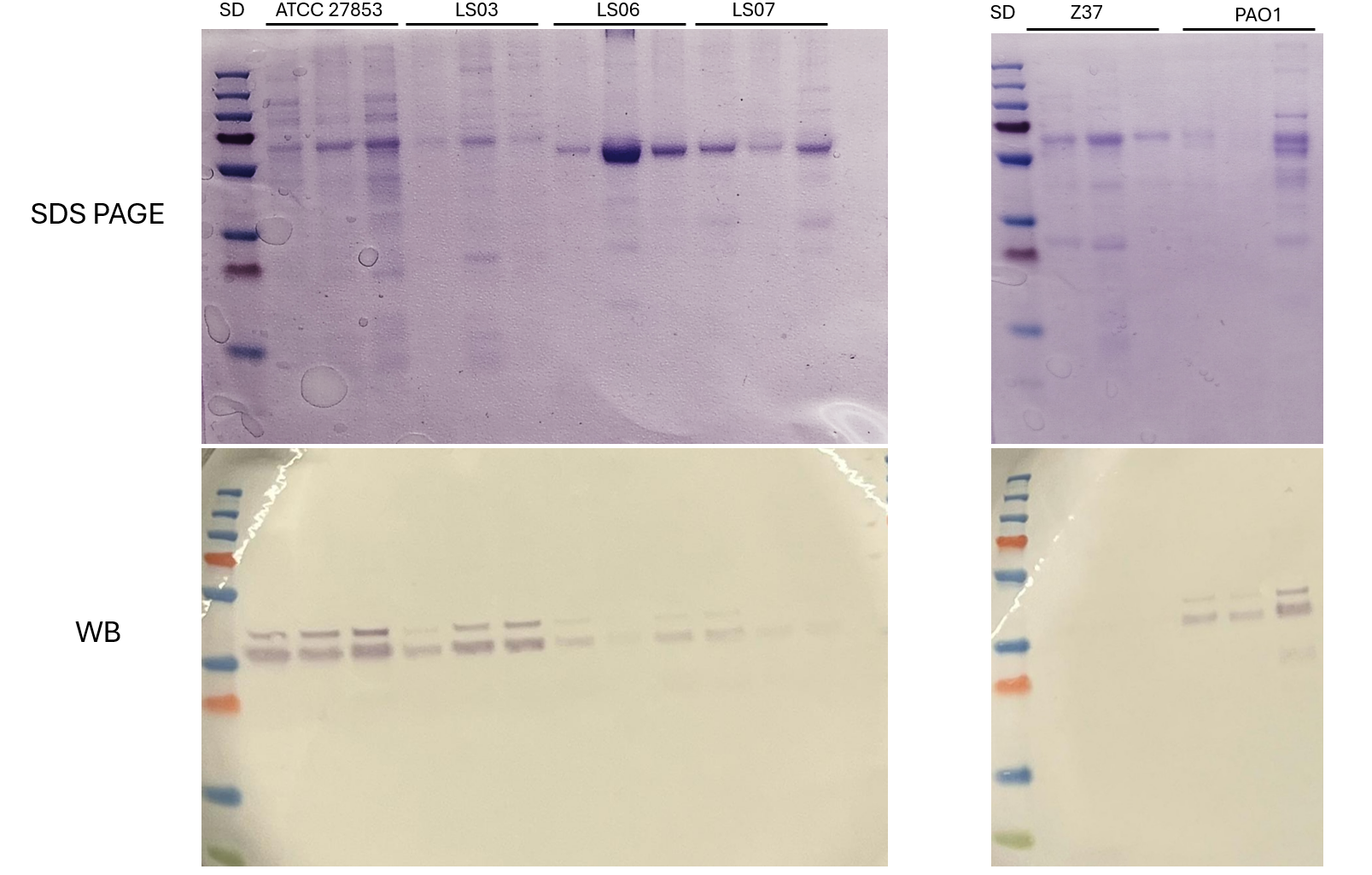


Figure S3. SDS PAGE and western blot of vesicle samples in triplicate.

Figure S1. SDS PAGE and western blot of sample in triplicate


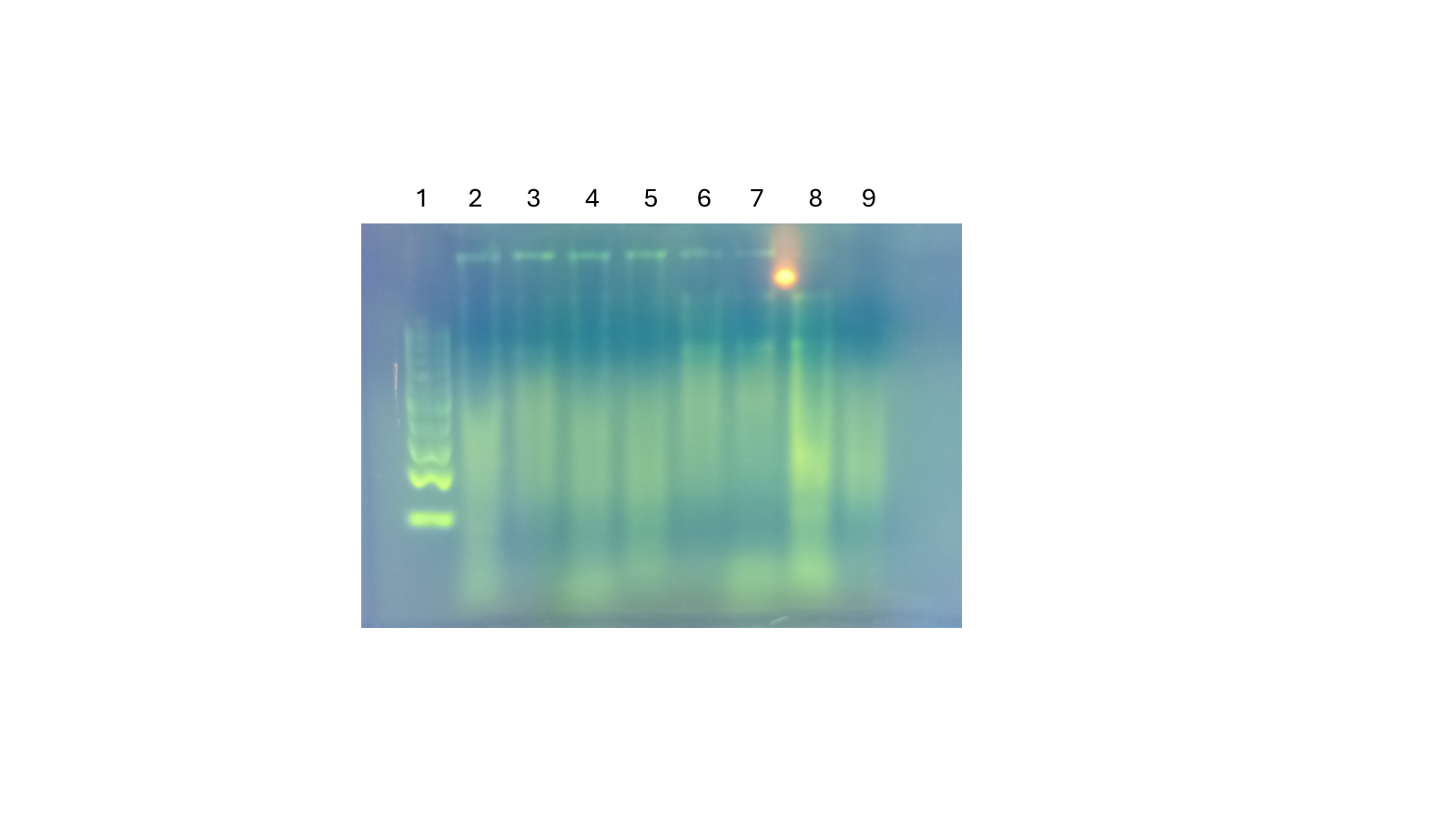


**Figure S4.** Agarose electrophoresis of vesicle samples. Extracellular vesicles were used as a sample for a 2% agarose gel electrophoresis with 1 × GelStar dye. 1: DNA marker; 2: PAO1 (pilot experiment); 3: PAO1; 4: ATCC 27853; 5: LS03; 6: LS6; 7: LS07; 8: Z37; 9: Z37 after DNase treatment.


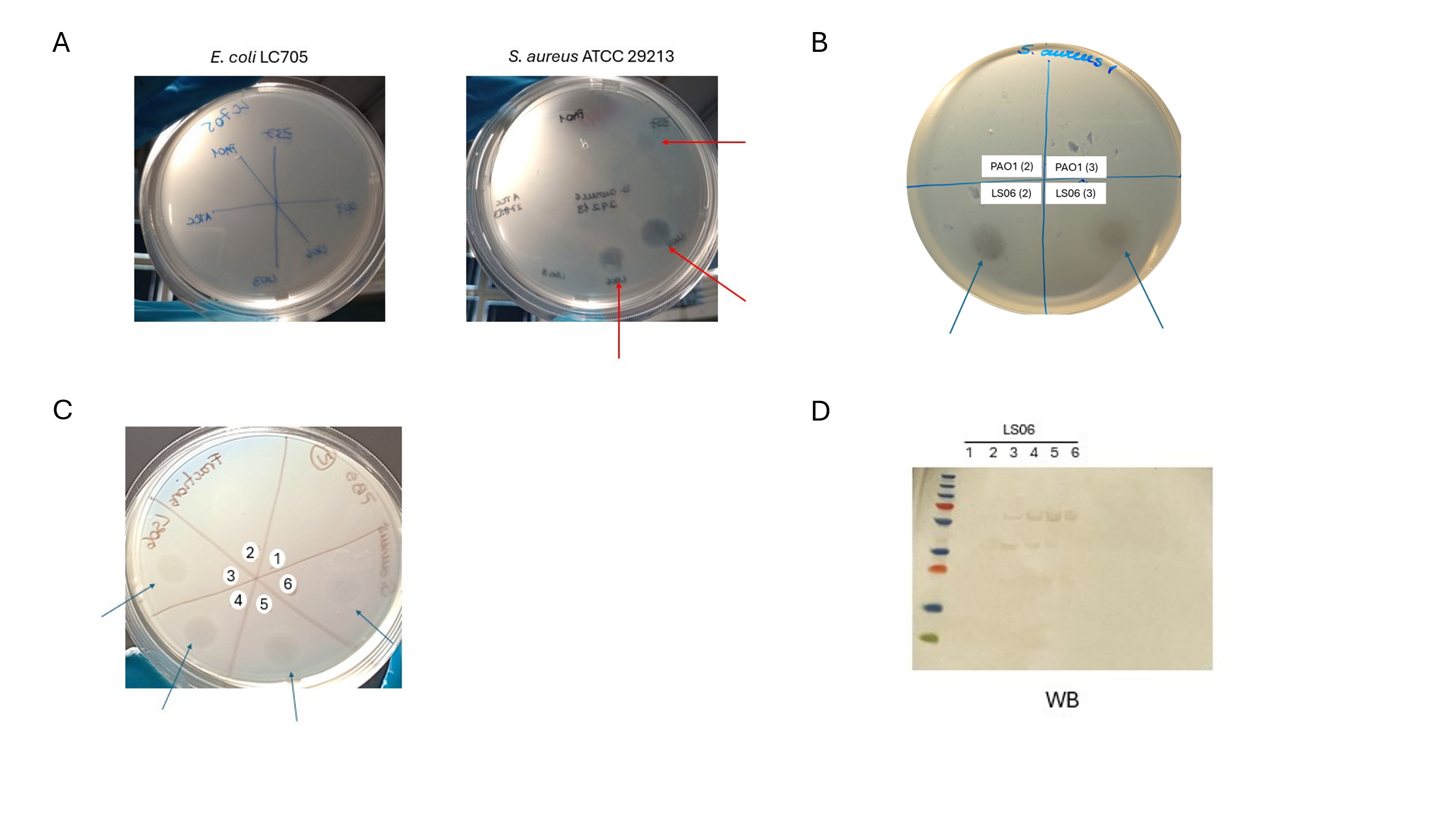


Figure S5. Bacteriolytic activity of vesicle extracts from clinical strains. A) Samples were tested against *E. coli* LC705 strain (left) and *S. aureus* ATCC 27853 (right). Red arrows on the right indicate inhibition zones generated by LS06, LS07 and Z37 extracts. B) Analysis of the bacteriolytic activity of PAO1 and LS06 vesicle extracts, replicates 2 and 3. Blue arrows indicate inhibition zones. Analysis of the bacteriolytic activity of LS06 replicates and fractions: C) Analysis of the bacteriolytic activity of LS06 fractions and D) Western blot of LS06 fractions using antibody against OprF. Blue arrows indicate inhibition zones. The numbers in Figure 5C correspond to the fractions loaded onto the gel in Figure 5D.
